# Socio-economic, built environment, and mobility conditions associated with crime: a study of multiple cities

**DOI:** 10.1038/s41598-020-70808-2

**Published:** 2020-08-17

**Authors:** Marco De Nadai, Yanyan Xu, Emmanuel Letouzé, Marta C. González, Bruno Lepri

**Affiliations:** 1 Department of Information Engineering and Computer Science, University of Trento, Via Sommarive, 9I, 38123 Povo, TN Italy; 2 Mobs Lab, Fondazione Bruno Kessler, Via Sommarive 18, 38123 Povo, TN Italy; 3 Department of City and Regional Planning and Department of Civil and Environmental Engineering, University of California Berkeley, 230 Wurster Hall #1820, Berkeley, CA 94720–1820 USA; 4 Data-pop Alliance, 99 Madison Avenue, New York, NY 10016 USA; 5 Department of Civil and Environmental Engineering, Massachusetts Institute of Technology, 77 Massachusetts Ave, Cambridge, MA 02139 USA

**Keywords:** Computational science, Statistics, Computer science

## Abstract

Nowadays, 23% of the world population lives in multi-million cities. In these metropolises, criminal activity is much higher and violent than in either small cities or rural areas. Thus, understanding what factors influence urban crime in big cities is a pressing need. Seminal studies analyse crime records through historical panel data or analysis of historical patterns combined with ecological factor and exploratory mapping. More recently, machine learning methods have provided informed crime prediction over time. However, previous studies have focused on a single city at a time, considering only a limited number of factors (such as socio-economical characteristics) and often at large in a single city. Hence, our understanding of the factors influencing crime across cultures and cities is very limited. Here we propose a Bayesian model to explore how violent and property crimes are related not only to socio-economic factors but also to the built environmental (e.g. land use) and mobility characteristics of neighbourhoods. To that end, we analyse crime at small areas and integrate multiple open data sources with mobile phone traces to compare how the different factors correlate with crime in diverse cities, namely Boston, Bogotá, Los Angeles and Chicago. We find that the combined use of socio-economic conditions, mobility information and physical characteristics of the neighbourhood effectively explain the emergence of crime, and improve the performance of the traditional approaches. However, we show that the socio-ecological factors of neighbourhoods relate to crime very differently from one city to another. Thus there is clearly no “one fits all” model.

## Introduction

Criminology widely recognizes the importance of places^1,2^: crime occurs in small areas such as street segments, buildings or parks, and it is spatially stable over time^3,4^. However, theoretical and empirical research showed that crime is also a consequence of socio-economic contextual characteristics, usually referred to as the “neighbourhood effect”^5,6^. In criminology, cooperation, as opposed to disorganization of neighbours, is indeed believed to create the mechanisms by which residents themselves achieve guardianship and public order^7^, solve common problems, and reduce violence^7–9^. This mechanism also finds its roots in urban planning, where the relationship between specific aspects of urban architecture^10^ and urban physical characteristics^11^ are related to security. Places and neighbourhoods are not to be considered islands unto themselves, as they are embedded in a city-wide system of social interactions. On a daily basis, people’s routine exposes residents to different conditions, possibilities^12^, and this routine may favour crime^13^. Nevertheless, many empirical studies focus on just a subset of static factors at a time such as socio-economic factors without considering the contextual built environment^8,9,14–17^, or ignoring mobility^15,16,18,19^, and often only drawing results in a single city (e.g. Chicago)^8,9,15,19–26^.

Studies on small areas and neighbourhoods roughly come from two streams of literature. The first stream focuses on the routine activity and crime pattern theories^13,27,28^ at places. These studies suggest that crime occurs when an offender, its suitable target, and the absence of any deterrence system, such as police or even ordinary citizens^29^, converge at a place. The presence of people influence the number of offenders and targets, but the daily routine of residents exposes homes and people to predatory crimes^30^. The built environment was also found to affect criminal activities, as physical disorder and specific locations (e.g. bar, taverns) attract offenders and suitable targets^31–33^. The second stream of literature builds on the social context upon which the place of the crime is embedded. A notable example is the Social Disorganization theory^7,9^, which found high crime concentration in socially and economically disadvantaged neighbourhoods. In it, the structural predictors are often seen through the concentrated disadvantage, ethnic diversity, residential instability of neighbourhoods^7,9,34^ While most of these studies use census data as primary data source, recent years have witnessed a growing interest in alternative data. For example, scholars exploited synthetic social ties to simulate neighbourhood cohesion^35^, and mobility flows to indicate crime opportunities and connections between neighbourhoods^23^. Others leveraged crowd-sourced Point of Interests (POIs), taxi flows^36^, and dynamic population mapping from satellite imagery^17,37^ and mobile phone activity^14,20^ to assess the presence of people. Altogether, these results highlight the tight relation between the socio-economic, the built environment and mobility conditions, and their impact on criminal activities. Although the two streams of the theory are often seen as competing, we argue that they can complement each other. However, very limited work has integrated socio-economic, built environment and mobility conditions together in multiple cities and in small areas. Moreover, while crime theories are not limited to specific cities^5^, and several cross-disciplinary results suggest common and universal patterns in mobility^38,39^, urban environment^40^ and aggregated crime^41,42^ in urban systems, our comparative knowledge base is limited^5^. These limitations result in a fragmented and incomplete picture^5,43^ of how the numerous factors influence crime in the urban context and limit the impact of the conclusions.

Here, we seek to shed light on the diverse set of factors at play with urban crime exploring how violent and property crimes are related, at the same time, to the Social Disorganisation, to the built environment characteristics and to human mobility. Specifically, we analyse crime at the level of group of blocks (measuring on average 0.378 square kilometers), considering both the local features of the group and its surrounding context, represented by all the blocks within a half-mile. The contribution of this paper is twofold. First, we address the need for a comprehensive study that explores crime patterns at fine grained resolution across multiple cities of the world, analysing Bogotá, Boston, Los Angeles and Chicago. Secondly, we show that taking into account the complex interplay between crime, people, places, and human mobility significantly improves the performance of the crime inference. We make use of massive and ubiquitous data sources such as mobile phone records and geographical data, implying that the resulting framework can be replicated at scale. Our generated insights can help recommend effective policies and interventions that improve urban security.

## Results

We study criminal activity in Bogotá (Colombia), Boston (USA), Chicago (USA) and Los Angeles (USA), four very different cities with respect to cultural, urban and socio-economic conditions.

**Figure 1:**
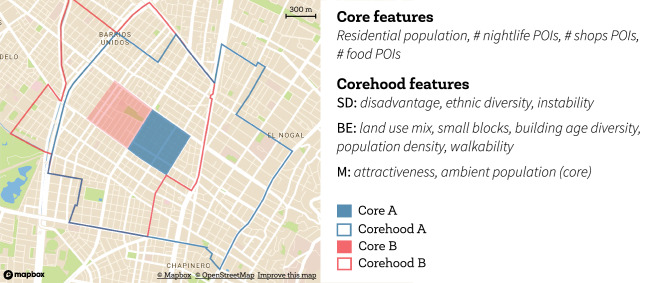
For each block group (the core), we consider the block groups within a half mile as its corehood. Blocks that are near each other share most of their corehood. In this example, we show two cores in Bogotá and their corresponding corehood. We focus on three aspects of the core and the corehood: the Social Disorganization (SD), the Built Environment (BE), and the Mobility (M). The core, where crime is predicted, measures on average 0.378 square kilometers.

Our approach follows the aforementioned two streams of literature of place and neighborhood, assuming the existence of a social process named neighborhood effect, namely the relation of crime patterns with small places characteristics and mobility. To account for all these factors we analyse criminal activity and small places characteristics at census block group, the smallest geographical unit for which the U.S. census publishes data, and measuring on average 0.378 square kilometers. Each block group, here called *core*, is exposed to a surrounding context, named *corehood*, which is the set of all the surrounding block groups within a half mile from the core (see Figure 1). As the context of nearby cores is similar, corehoods might overlap. The idea of using overlapping units is not new^16,44,45^, and it is focused on creating an ego-centric neighborhood for each core (see Supplementary Information (SI) Note 11 for a technical discussion). We describe the characteristics of the place where crime happens through specific features of the *core*, while we describe the context at which it is embedded through the features at the *corehood*. As neighborhoods in literature are loosely defined, we tested different sizes of the corehood, finding the half mile distance as the best to describe the neighborhood effect (see the SI Note 11).

Criminal activity is provided by police agencies, which record through police reports the geographic location (i.e. latitude and longitude), date, time of day and category of each crime event. For all the cities we map each category of crime into the US Uniform Crime Reporting (UCR) categories^46^ and analyse crime belonging to two broad categories: violent and property crimes. They include homicides, sexual and non-sexual aggravated assaults, robbery, motor vehicle thefts and arson. We assign each crime to a corehood through its geographical position.

We describe cores through the features that were previously found to attract potential offenders and targets^36^, such as the census *residential population* and the number of *nightlife*, *shops* and *food* POIs inside each core, which are extracted from web data (more details in the Methods Section). Then, we describe corehoods through the environmental (neighbourhood) characteristics found to influence crime^11,24^. The corehood features are estimated from all the block groups surrounding the core. We group them in Social Disorganization (SD), Built Environment (BE) and Mobility (M) features. The SD characteristics include some of the standard Social Disorganization theory features, namely concentrated *disadvantage*, *instability* and *ethnic diversity*. Consistently with the literature^7,9,15,26^, *disadvantage* and *instability* are composite variables built from the two largest principal components of: (i) unemployment rate, (ii) poverty rate, defined as people living below the poverty line, and (iii) residential mobility rate, defined as the percentage of people who recently changed residency. Again, in accordance with the literature^7,47,48^, *ethnic diversity* is computed as the Hirschman-Herfindahl index across six population groups (e.g. hispanic, black, white people, etc.). Additional details are present in the Methods Section. Note that we excluded all race-specific variables that are usually employed (e.g. percentage of black people in^36^) to build an evidence-based and race-neutral model.

The BE corehood features are based on the Jane Jacobs theory^11^, which states that the presence of people and a vibrant neighborhood life form a virtuous loop controlling local crime. From her own words “a well-used city street is apt to be a safe street and a deserted city street is apt to be unsafe”^11^. Four conditions have to be valid to ensure this virtuous loop. First, a district should serve at least two or more functions to have streets continuously used by residents and strangers. Second, street blocks should be small and short to ensure both high *walkability* and frequent meeting of people at street intersections. Third, diverse buildings make it possible to have low- and high-rent spaces, and thus a mixture of people and enterprises. The fourth condition is about dense concentration, which ensures a sufficient presence of people and enterprises to attract dwellers from different neighbourhoods continuously. Thus, in accordance with the literature^49^ we operationalize through census and geographical data the four conditions in: i) *land-use mix*; ii) *block size* iii) *building age diversity*; iv) *population density* and *walkability*, which promotes social relations^50^ and is connected to local cohesion of neighbors. The details, data sources, and formula for these metrics are available in the Methods Section.

The M features are built upon recent mobility and criminology literature, which found mobility to be tightly coupled with criminal activity in space and time^14,20,25,42^. People at risk in urban areas can be essentially measured through residential and floating population. The first one measures the number of people who resides in an area, while the second one measures the average number of people that can be expected in an area at any given time^37^ (e.g. average number of people at a mall). We measure floating population through the average number of people for each core, named *ambient population*^37^, and the *attractiveness* of the corehood, measured through the number of people movements to the corehood for reasons different than travelling to work or home. We extract this valuable information from passively and anonimized mobile phone data, collected by mobile phone operators for billing reasons. From mobile phone data, we fit the mobility model TimeGeo^51^ and simulate realistic urban traces that are used to extract the *ambient population* and *attractiveness* features. We do not include M features for Chicago, as we do not have mobile phone traces. Even if mobility is not available, Chicago is considered by many the testbed for empirical crime analysis, thus we include it to allow readers to do useful comparisons for socio-economic and urban environment characteristics.

Crime patterns have been observed to be highly concentrated in the space, overdispersed^52^, and positively spatial correlated. Thus, we model and predict crime through a spatially filtered Bayesian Negative Binomial, which is specifically tailored for discrete data, accounts for the overdispersion of crime events, models uncertainty and avoids the biased parameters of non-spatial models^53,54^. Through this model, criminal activity at cores is described by a linear combination of an intercept, the fixed effects (i.e. the aforementioned core and corehood features), and some random effects that represent the latent and unexplained variance that emerge from the spatial-autocorrelation of neighboring areas. Our model accounts for the high spatial correlation in crime events, and we did not find any significant spatial auto-correlation in the model residuals (see Note 4 in the SI). The reader can refer to the Methods section for additional details about the model and its formulation.

**Table 1:** Quantitative results of crime description and predictions in Bogotá, Boston, Los Angeles and Chicago. The model including Social Disorganization, Built Environment and Mobility features achieves the highest descriptive ($R^2_m$ and $R^2_c$) and predictive (LOO) performance. Here, we can see that contextual features of the neighborhood significantly increase our model’s performance against the model considering only the core features. The LOO metric is calculated through the Pareto smoothed importance sampling Leave-One-Out cross-validation.

Model	Bogotá		Boston		Los Angeles		Chicago	
	$R^2_m$ ($R^2_c$)	LOO	$R^2_m$ ($R^2_c$)	LOO	$R^2_m$ ($R^2_c$)	LOO	$R^2_m$ ($R^2_c$)	LOO
Core	0.54 (0.75)	−3897	0.21 (0.64)	−2035	0.18 (0.68)	−9665	0.09 (0.68)	−8415
Social-disorganization (SD)	0.57 (0.75)	−3891	0.55 (0.68)	−2019	0.53 (0.72)	−9529	0.66 (0.78)	−8019
Built environment (BE)	0.61 (0.76)	−3881	0.36 (0.68)	−2014	0.27 (0.69)	−9629	0.21 (0.69)	−8371
Mobility (M)	0.64 (0.80)	−3804	0.42 (0.70)	−2001	0.25 (0.70)	−9570	-	-
SD+BE	0.64 (0.76)	−3881	0.65 (0.72)	−1987	0.56 (0.72)	−9508	$\mathbf{0.67\ (0.79) }$	$-\mathbf{8003 }$
SD+M	0.66 (0.81)	$-\mathbf{3795 }$	0.67 (0.73)	−1973	0.55 (0.73)	−9467	-	-
BE+M	0.68 (0.80)	−3819	0.50 (0.72)	−1989	0.30 (0.70)	−9585	-	-
SD+BE+M (Full)	$\mathbf{0.70\, (0.80) }$	−3808	$\mathbf{0.70\, (0.75) }$	$-\mathbf{1957 }$	$\mathbf{0.56\, (0.74) }$	$-\mathbf{9454 }$	-	-

The best performance is highlighted in bold.

### Description and prediction of crime

We begin by presenting the aggregated performance of our model predicting crime in the four analysed cities. We evaluate our model under various feature combinations to assess the contribution of each group of features. We measure the capability of the model to describe crime through the marginal $R^2_m$^55^ and the conditional $R^2_c$^55^ (the higher the better). The marginal $R^2_m$ measures the proportion of variance explained by the fixed effects (i.e. the input features), while the conditional $R^2_c$^55^ takes also into account the variance explained by the auto-correlation but not the input features (absorbed by the random effects). The difference between the two can be used to find clustering effects and missing variables. To assess the point-wise out-of-sample prediction accuracy we use the Pareto-smoothed importance sampling Leave-One-Out cross-validation (here called LOO for simplicity)^56^ (the higher, the better).

First, we evaluate the baseline model that includes only the core variables, namely the residential population and the number of nightlife, shops and food POIs. Table 1 shows that the core-only model performs poorly in Chicago, Los Angeles and Boston, while it has high $R^2_m$ in Bogotá. We observe high difference between $R^2_m$ and $R^2_c$, which means that there is a significant unexplained variance that is not explained by the core features.

The SD, BE and M features significantly increase the explanatory power of our model. Particularly, in US cities, the $R^2_m$ increases up to 161%, 194% and 633% in Boston, Los Angeles and Chicago. Notably, and not surprisingly, the SD features are very important, especially in Chicago, where the “Chicago school”^57^ forged the Social Disorganization theory and further elaborated the role of collective efficacy on dealing with crime. Differently, the increase in Bogotá is less pronounced, suggesting that the neighbourhood impact on crime is limited. Turning to M and BE features, we find that they describe the crime, but they are often as not meaningful as the SD features for crime prediction. However, the importance of mobility confirms the importance of floating population at describing micro-dynamic behaviour of criminal activity^25,42^. We observe that in all cities the conditional $R^2_c$ increases when adding the SD, BE and M features, revealing that the included variables also help explain the variance of crime.

Overall, Table 1 shows that considering together SD, BE and M variables result in the highest descriptive ($R^2_m$) and predictive (LOO) performance. This result means that, in order to model crime, one needs to account for multiple aspects of urban life, including Social Disorganization, the physical characteristics of the neighbourhoods, and mobility. This result holds also against different combinations of the features (i.e. SD+BE, SD+M and BE+M). Nonetheless, some of the SD+BE and SD+M models are very competitive and might be considered when all data-sources are available. Particularly, the ambient population (i.e. the average number of people who stop at the core) is one of the most important variables in the model and allows to better assess the number of people at risk, as suggested by previous works on aggregated mobility^42^, satellite imagery^37^, Twitter^20^ and census data^58^. The $R^2_m$ improvements also indicate that the model relies less on the random effects and it is better at explaining crime from the input features. However, we found that it might generate large errors due to places that are outliers of mobility in densely populated areas or hotspots of activity (see Figure S16 and Figure S17 in the SI).

**Figure 2:**
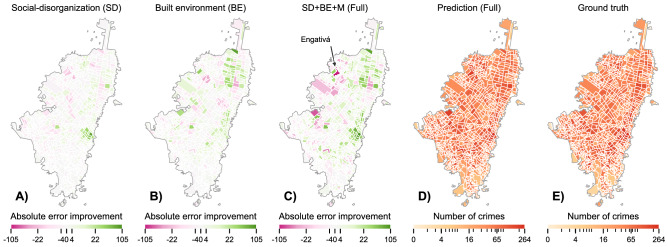
Maps of the estimated number of crime for each neighborhood in Bogotá for the A) Social-disorganization, B) Built environment, C) Full model. D) shows the Full model’s prediction. E) shows the ground truth crime count.

Figure 2 shows the spatial gain in performance from the baseline in Bogotá. First, it reveals that our Full model prediction resembles the ground truth data (Figure 2 D-E), as confirmed by the high value of $R^2_c = 0.80$. Second, it shows that, while the SD and BE models achieve localized improvements (Figure 2 A-B), the Full model improves the prediction almost everywhere. However, the Full model performs quite poorly in a specific area of Bogotá (see Figure 2 C), part of the Engativá neighbourhood. By inspecting the coefficients of the model, we find that this area is an outlier as it is densely populated, thus causing an inflated prediction of crime, due to the high importance of residential and ambient population in the Bogotá model. Note, however, that our prediction is at the block level and the city-wide goodness of fit is $R^2_c = 0.80$.

The difference between $R^2_c$ and $R^2_m$ represents the unexplained variance due to spatial auto-correlation, which might suggest missing effects and variables. In Bogotá, our model points out that the touristic and dangerous neighbourhood La Candelaria, and the populous district of Engativá have significant unexplained variance that our input features cannot capture (see Figure S13 in the SI). In Boston, the area near the Franklin park indicates missing local factors (see Figure S12 in SI). In Los Angeles, unexplained variance seems to be tied to places with a large number of people, namely the international airport and the UCLA campus (see Figure S14 in SI). Again, in Chicago, missing variables are suggested near the prison and the southern area (see Figure S15 in SI). Altogether, these signals could help policymakers on including the best factors for each city and enacting policies that prevent crime.

Previous results suggested that human movements between different regions might help describing crime^36,59^. Thus, we test our model against this hypothesis by using the people trips between areas to model the auto-correlation between corehoods. This connectivity is not only influenced by distance but also by geographical barriers, roads, traffic, and public transportation. Moreover, it could be interpreted as a proxy of spatial mismatch and isolation, which was empirically found to be connected with crime^60^. To build the connectivity matrix we use the TimeGeo model, which simulates a reliable Origin-Destination matrix between regions and it is validated towards transportation surveys (see Supplementary Note 3.1). However, we find that mobility flows alone do not have good predictive power in LA and Boston. The interested reader can find more information on the definition and results of this connectivity matrix in Supplementary Note 6.

While the effects of urban environment characteristics, socio-economic conditions, and mobility have been empirically tested separately^9,49,60–62^, to the best of our knowledge, this is the first study to support with large-scale data the association of crime with socio-economic conditions, the built environment, and mobility. However, we find that these aspects do not play the same role across cities, and only some of them contribute to the crime prediction model.

**Figure 3:**
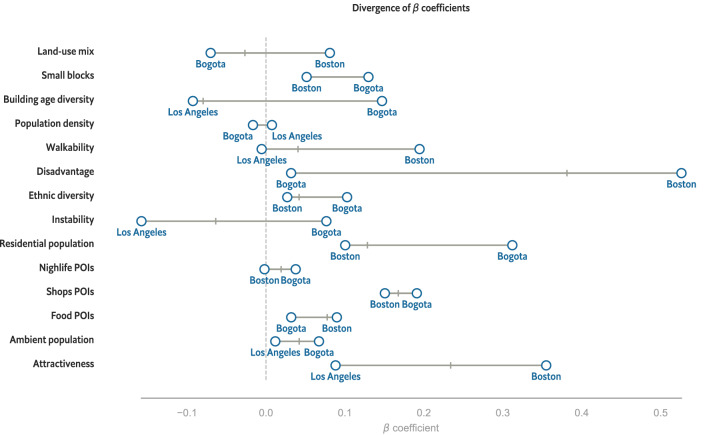
Generalized Linear Model’s $\beta$ coefficients showing that Social Disorganization, Built Environment and Mobility features do not play the same role in all cities. We highlight in blue the minimum and maximum coefficient for each feature. Overall, this figure shows that there is no universal theory of crime for spatial predictions.

### Neighborhood variables across cities

By comparing how features play different roles in different cities, we can understand how far can we push previous theoretical and empirical studies. In this section, we turn our attention to the standardized ${\beta }$ coefficients that reveal how features correlate with criminal activity.

First, we focus on the coefficients of the Full model, which combines socio-economic features with the characteristics of the built environment and human mobility. Note that here Chicago is excluded for lack of data. Figure 3 pictures that the ${\beta }$ coefficients vary greatly across cities. For example, land-use mix correlates negatively with criminal activity in Bogotá and Los Angeles, but positively in Boston. Similarly, higher population building age diversity is present in low-crime areas in Boston and Los Angeles, but in high-crime areas in Bogotá. Social Disorganization variables are no less different, as corehood instability is correlated with crime activity only in Bogotá, differently from what expected from the theory^7,63^.

The discrepancies between cities could be explained by the different spatial and socio-economic processes at play. When we look at the bivariate correlations across features, we observe interesting patterns. For example, in Los Angeles and Boston, *walkability* is strongly positively correlated with population density and neighbourhood attractiveness, as expected^7,63^, and slightly correlated with advantaged neighbourhoods. Differently, walkable areas in Bogotá have low population density and are highly advantaged, while the attractiveness is slightly correlated (see Figure S20 in SI). A possible reason for the ${\beta }$ coefficients disagreement lies on the multi-collinearity of the input features. Although we use the QR decomposition and Ridge penalty to shrink down the variables that are not necessary, the difference between the coefficients is present also in simpler models (e.g. core-only).

The difference between the results across cities also suggests that crime correlates differently with space and people. For example, we observe that in Bogotá high crime areas relate to advantaged neighbourhoods, while in Boston and Los Angeles higher crime seem to be linked to disadvantaged neighbourhoods, according to the theory^7,63^. A possible explanation might be related to under-reporting and police disrespecting, which seems to be a problem particularly in Bogotá^64^. However, literature has shown how neighbourhood cultural codes, informal local control, and problematic policing are also related to violent criminal activities^15^.

We also found some commonalities in all the cities. We find that corehoods with high disadvantage and ethnic diversity but, surprisingly, smaller blocks have higher crime activity. While in the core we find that the presence of Shops, Food POIs, and population (both residential and ambient) correlates positively with criminal activity. These results resonate with literature showing that the presence of POIs and ambient population increase crime due to a higher number of potential targets and offenders in an area. Additionally, we find that corehood attractiveness has a strong connection with crimes, suggesting that the presence of people that do not live nor work in the area might influence crime. This result is in contrast with literature based on Jacobs’ theory^11,22^, but resonate with Oscar Newman’s one arguing that a high number of visitors results in higher anonymity and, thus, crime^10^. Additionally, a recent empirical study from survey data^65^ agrees with our result, obtained instead with large-scale and passively collected information. In the supplementary materials (SI), we compare all the cities in detail (see Supplementary Note 5-11).

We acknowledge the big difference between crime types. In this paper, we analysed serious crimes, which comprise heterogeneous crime types such as rape and robberies. Thus, we also test our model by disentangling criminal activity into two main categories: property and violent crimes. We found that the Full model still outperforms the others, and that precise patterns can be extracted from the $\beta$ coefficients analysis. For example, in Bogota *walkability* is much more important in describing property crime than violent crime, while in Los Angeles, higher *walkability* seems to suggest a lower presence of property crimes. However, we observe that the multifaceted picture found in the aggregated crimes still holds for the disentangled models.

We also tested the alternative assumption where all corehood features are computed at the core, and found that the models with features computed at the corehood perform better than the models using SD, BE and M features only at the core, which highlights the validity of the corehood (and neighbourhood) assumptions (see Supplementary Note 11).

Previous research have found universal common patterns even in highly heterogeneous data and behaviour. Literature has shown the existence of common mathematical models describing mobility^38,39^, cities^40^ and aggregated crime at the city level^41,42^. To test the possibility of having a universal model that predicts crime in small areas, we test a model that uses only the features that behave in the same direction in all the cities. This model consistently performs worse than the Full model (see Note 10 in SI), showing that at this moment, no model is convenient to be easily applied to all cities. We also studied at what extent a model trained in one city can be tested to another city. We found that US cities are, as expected, more similar to each other than Bogotá, and that Los Angeles behave similarly to Chicago.

## Discussion

In this paper, we modelled the presence of crime across four cities, widely different with respect to cultural, economic, historical and geographical aspects. We found that the variability of the dynamics and history of each city poses a challenge to the existence of a model that “fits it all”, able to learn from one city and to predict on another one. Instead, we presented a model that could describe and disentangle the role of diverse factors in urban crime and draw some theoretical and practical implications.

The goal of this research goes beyond crime prediction in time (i.e. forecasting). Offences are concentrated in a small number of places^66^, and are tightly coupled with places, stable over time^1^. Thus, the easiest way to predict crime is modelling those few places with the highest number of crimes, also known as *hotspots*^14,67^. On the contrary, we seek to shed light on the diverse set of factors at play with urban crime and do predictions for those areas without crime statistics (i.e. nowcasting).

Our cumulative results show little evidence in support of the Jane Jacobs’ theory, arguing that specific urban features and people on the street generate higher security. On the contrary, we often found that Jacobs’ features and urban vibrancy increase people’s vulnerability to crime, suggesting that further work has to be done in this direction.

We found that different theories often seen as competing can complement each other in models that take into account the socio-economic, built environment and mobility conditions together. The importance of mobility and built environment characteristics showed that competitive descriptive and predictive models can be built from data available at large scale without the necessity of costly in-field survey studies. However, we found that aspects related to the Social Disorganisation are important for crime description and prediction. Therefore, it is crucial to consider alternative sources of data to infer social cohesion and interactions and overcome the use of census information, which is costly to collect and rarely updated. There have been multiple attempts at inferring social interactions^68^, poverty^69^, well-being^70^ and unemployment^71^ but so far very little work has been done at small areas.

Comparing multiple cities in different countries do not come without limitations. First, our analysis ignore temporal variation such as opening times of POIs or temporal variation in mobility. Second, due to lack of consistent data, we did not account for variables such as political and housing policies, security perception, community participation, and social ties within family and within neighbourhoods that were previously found to be related to crime^33,72,73^. Finally, official crime data do not come without errors, given that not all crimes are reported nor recorded^74^, and there is no “ground truth” data to gauge any bias in police records. We use official police records similarly to recent literature in the field^14,16,20,25,36^.

Our work seeks to make headway on the previous limitation of a single site of study. While recent works have started the use of street units and blocks to study criminal activity^19,21,75,76^, they often relied on a small subset of variables and one city. Analysing multiple cities together exposed criminology theories to discrepancies and differences, and answers to the call of a framework to compare crime in different cities^43^. Descriptive and comparative modelling can help policymakers to see common patterns between cities, understand the use of urban space and deploy future investments and resources thoughtfully. Moreover, from the scientific perspective, descriptive modelling can provide insights for strong predictors, and potentially for explanatory variables, to be further investigated by explanatory modelling and experiments^77^. Thus, we hope that additional research keeps exploring multi-dimensional aspects related to crime, to clarify potential crime causes and design better cities.

## Methods

The socio-economical and Jane Jacobs’ urban theories are dependent upon the actions and activities at work in communities. Thus, we identified corehoods as social and geographical units of analysis. Then, we obtained and aggregated the data for each corehood of Bogotá, Boston, Los Angeles and Chicago.

### Crime data

Our crime data is obtained directly from police departments. Crime records are collected by the police, which annotates in the report the crime event at point locations (latitude and longitude) along with the category of crime and the time it happened.

Through its category, we associate each event to the Uniform Crime Reporting (UCR)^46^ categorization. The UCR program is a US statistical effort to make crime reports uniform across the country. The UCR divides crime in two main groups: Part 1 and Part 2 offences. The former is composed by violent crimes (aggravated assault, forcible rape, robbery and murder) and property crimes (larceny-theft, motor vehicle theft, burglary and arson), while the latter are considered less serious and they include offences such as simple assaults and nuisance crimes.

We filter out those crimes not belonging to Part 1 of UCR, similarly to most of the criminology literature. For Bogotá we mapped crime categories consistently with UCR categories, and we released the mapping for future research and comparisons. We also filtered out larceny crime events, which include among others thefts of bicycles, shoplifting, pick-pocketing, or the stealing of any property or article that is not taken by force and violence or by fraud. We consider larceny-thefts (except motor vehicle theft) as sometimes noisy and we expect the neighborhood effect to have a negligible impact on larceny-thefts (e.g. social cohesion with pick-pocketing in a shop). We geo-reference crimes to cores and, when a crime event happens in a street segment shared between cores, we evenly assign the event to both cores. Due to the limit in accuracy of GPS positioning, we create a buffer of 30 meters for each crime, which is the distance usually employed for stop location detection algorithms^78^ and criminology literature at micro-places^21,44,76^. We have no reason to suspect that the effect of the crime events stops at distances lower than 30 meters (e.g. robberies on the other side of the street are likely to affect residents on both sides). On the contrary, crime risk at hotspots has been observed to spread to distances up to 2000 meters^67^ spatially. Moreover, we note that the median area of cores are 0.378 square kilometers, which roughly means that each core has a median side of 615 meters (see Figure S11 in the SI).

More details are presented in the SI. We summed crime events over one year to minimize seasonal fluctuations.

### Mobile phone data

We computed the ambient population and the OD matrices for Bogotá, Boston and Los Angeles from Call Detail Records (CDRs) of millions of individuals in the three cities. Mobile phone activity includes received and made calls and SMS activity. Each time a call or SMS is made/received, a CDR is generated. It includes some metadata such as the time and the tower at which the phone was connected when the activity was collected. Due to the inherent noise of CDRs^79^, which are collected only for billing purposes, we follow seminal literature^78,80,81^ and apply a stop location algorithm to classify the geo-located points where people *stay* or *pass-by*. Then, we simulate reliable human mobility traces through the TimeGeo modelling framework^51^, which generates traces that well describe the real mobility of people. To be consistent with the travel surveys of each city it simulates the time, duration, direction and type of travels within the city. The types of travels are classified as Home-Based from/to Work (HBW), Home-Based from/to Other type of locations (HBO) and Non-Home-based from/to Other type of locations (NHB).

We fitted the model starting from aggregated and anonymized Call Detailed Records (CDRs) collected from 12-01-2013 to 05-31-2014, 6 weeks in 2010, and 10-15, 2012 to 11-24, 2012 for Bogotá, Boston and Los Angeles respectively. We validated the model with the National Household Travel Survey (NHTS)^82^ and California Household Travel Survey (CHTS)^83^ datasets. We refer to the SI for the validation of TimeGeo.

To build the *ambient population* we counted the number of people who stops at a specific location for at least one hour. Since TimeGeo is validated and peer reviewed with HBW, HBO and NHB types of trips, we define the corehood *attractiveness* counting the number of NHB trips with the corehood as destination. We did not use HBW trips, as we cannot differentiate the origin from the destination and thus attractiveness could correlate with residential places. For the same reason, we excluded HBO trips from the *attractiveness* definition.

The anonymized data for the three cities was collected for billing purposes by two mobile operators, who also kindly provided to us the data for the present research.

### Spatial and census data

Census blocks, population, employment and poverty for US cities were drawn from the American Community Survey (ACS) (https://www.census.gov/programs-surveys/acs). The census data of Bogotá was obtained by the Departmento Administrativo Nacional de Estadística (DANE), which organized the 2005 general census for the city (http://www.dane.gov.co). The poverty data of Bogotá was extracted from the Sisbén in the Identification System III of 2014. We also use the US Tiger dataset, OpenStreetMap (http://www.openstreetmap.org) geographical data and the POIs extracted from Foursquare (http://www.foursquare.com). The detailed description of datasets and related source URLs are listed in the SI.

### Built environment features

We operationalize the Jane Jacobs conditions through some state of the art metrics defined in literature^49^ in all the corehoods. The land-use mix is computed as the average entropy among land uses: $$\text {LUM}_{L,i} = - \sum _{j \in L} \frac{P_{i,j} \log (P_{i,j})}{\log (|L|)}$$, where $$P_{i,j}$$ is the percentage of square meters having land use *j* in unit *i*, and $$L = \{\text {residential}, \text {commercial and institutional},$$$$\text {park and recreational}\}$$ represents the considered land uses in the metric. The LUM ranges between 0, wherein the unit is composed by only one land use (e.g. residential), and 1, wherein developed area is equally shared among the *n* land-uses.

Then, for each corehood we determine the *walkability* through the accessibility of the core to the nearest point of interests (e.g. convenience stores, restaurants, sport facilities). Consistently with literature^84^, we define the weighted *walkability* score as: $\text {walk}_i = \frac{1}{|B_i|} \sum _{c \in C} \sum _{b \in B_i} {{\,{\mathrm{wdist}}\,}}(b, {{\,{\mathrm{closest}}\,}}(b, \text {POI}_c))$, where *C* is the set of categories (i.e. Food, Shops, Grocery, Schools, Entertainment, Parks and outside, Coffee, Banks, Books), ${{\,{\mathrm{wdist}}\,}}$ is the street-network distance decay function, and $\text {POI}_c$ is the set of POIs of category *c*. The distance decay function gives a weight (importance) to each POI reachable from a starting point. Additional information about the *walkability* score can be find in the SI.

We then compute the average block area among the set $B_i$ of blocks in unit *i* as $\text {Blocks area}_i = \frac{1}{|B_i|} \sum _{b \in B_i} {{\,{\mathrm{area}}\,}}(b)$, and the building age diversity as the standard deviation of building ages in the corehood.

Finally, we operationalize Jacobs’ density condition with the dwelling units density, computed from census data. Additional details are described in the SI.

### Social Disorganization

We create the feature *disadvantage* and *instability*^7,9,15,26^ through the two largest PCA principal components of: (i) unemployment rate, (ii) poverty rate, defined as people living below the poverty line, and (iii) residential mobility rate, defined as the percentage of people who recently changed residency (one year for US cities and fiver years for Bogotá). From the loadings of the PCA linear combination we verified that disadvantage is mainly a linear combination of poverty rate and unemployment, while instability is mainly about residential mobility rate.

In the Social Disorganization variables we do not include any ethnic-specific variables (e.g. percentage of black people) other than diversity because they might be present only in some places and not in others (e.g. native Americans in Bogotá), and to avoid any ethnic-specific bias. Ethnic diversity represents the difficulties of a community to communicate and collaborate for a common goal. Accordingly to the literature^7,47,48^, it is computed as the Hirschman-Herfindahl diversity index of six population groups $H = 1- \sum _{i=1}^N s_i^2$, where $s_i$ is the proportion of people belonging to the ethnicity *i*, and *N* is the number of ethnicities. Consistently with the literature we include for US cities: Hispanics, non-Hispanic Blacks, Whites, Asians, Native Hawaiians - Pacific Islanders and others. For Bogotá we include: Indigenous, Rom, Islanders (San Andrés), Palenquero, Black and others.

### Bayesian model

Let $y_i$ be the discrete number of crimes for a set of spatial regions $i= 1 ,\ldots , N$. We approximate the relation between crimes and spatial features through a Negative Binomial approach that models the non-negative nature of the crime-counts in a city, but also the overdispersion found in the data (Note 4 in the SI). Specifically, $\ln ({{\mathbb {E}}}(Y)) = {\mathbf {X}}\beta + {\mathbf {b}}$ where ${\mathbf {X}}$ is the input data and ${\beta }$ the coefficients of the model. ${\mathbf {b}}$ are the random effects that accounts for the unexplained variability of crime (i.e. the spatial-autocorrelation). In this paper, we account the spatial auto-correlation with the Bayesian Spatial Filtering (BSF)^85^ that defines ${\mathbf {b}} = {\mathbf {E}}{\gamma }$ where ${\gamma }$ are coefficients to be found. ${\mathbf {E}}$ is instead defined as the first principal components of ${\mathbf {E}}_{\mathrm{full}} = \mathbf {MCM}$, where ${\mathbf {C}}$ is a spatial matrix that describes the graph between spatial locations, while ${\mathbf {M}} = {\mathbf {I}} - {\mathbf {X}}({\mathbf {X}}'{\mathbf {X}})-{\mathbf {X}}'$, which is an approximation of the spatial error model^54^. We tested for the presence of spatial auto-correlation on the residuals of all the models without finding significant auto-correlation. As the results might change with different definitions of ${\mathbf {C}}$, we tested all the models for three definitions: i) ${\mathbf {C}}$ is a binary adjacency matrix identifying whether a corehood overlaps another corehood, ii) ${\mathbf {C}}$ is a inverse distance matrix between corehoods, iii) ${\mathbf {C}}$ describes the flow of people between corehoods, which is extracted from mobile phone data. We found that the binary matrix consistently outperforms other definitions. Additional details of the presented models, definition of ${\mathbf {C}}$, and other competitive models tested are present in the SI.

As we have to account for collinearity, we employ a Ridge penalty to all fixed effects.

### Model calibration ed evaluation

Model calibration is carried out by means of Markov Chain Monte Carlo (MCMC) approach. We run the MCMC method for 20,000 iterations and chose as burn-in the first 15,000 iterations to ensure that the remaining 5,000 iterations are in the high-probability region. Convergence for all the models was assured by the Gelman-Rubin convergence statistics^86^ and visual inspection of the traces.

We assess how well the models describe crime through the conditional $R^2$ and the marginal $R^2$^55^, which adapt the popular coefficient of determination to the generalized linear mixed-effects models. They are defined as:

$$\begin{aligned} R^2_m&= \frac{\sigma _f^2}{\sigma _f^2 + \sigma _r^2 + \sigma _{\epsilon }^2}\\ R^2_c&= \frac{\sigma _f^2 + \sigma _r^2}{\sigma _f^2 + \sigma _r^2 + \sigma _{\epsilon }^2} \end{aligned}$$

where $\sigma _f^2$ is the variance explained by the fixed effects, $\sigma _r^2$ is the variance explained by the random effects, and $\sigma _{\epsilon }^2$ is the variance of the residuals. Specifically, $f= {\mathbf {X}}\beta$, $r= {\mathbf {E}}\gamma$ and $\epsilon$ is specific to the Negative Binomial and defined^55^ as $\epsilon = \ln {(1+1/\mu +1/\phi )}$, with $\mu = \frac{1}{N} \sum _i^N y_i$ and $\phi$ is the shape parameter of the Negative Binomial distribution.

We assess the out of sample predictive accuracy through the Pareto-smoothed importance sampling Leave-One-Out cross-validation (PSIS-LOO, here simply referred as LOO)^56^ and the Deviance Information Criterion (DIC)^87^. Even though DIC has been used extensively for practical model comparison in many disciplines, recent literature on Bayesian models evaluation strongly discourage the use of DIC due to its numerous disadvantages including the fact that it works well only if the posterior is close to a Gaussian, its lack of consistency and the fact that is not a proper predictive criterion^56,88^. Since LOO overcome the DIC issues, it has rapidly become the state of the art for evaluating Bayesian models. We employ the LOO in the main paper, while we present the DIC results in the supplementary. The LOO is defined in the log score as:

1 $$\begin{aligned} \text {LOO} = \sum _{i=1}^n \ln \left( \frac{\sum _{s=1}^S w_i^s p(y_i|\theta ^s)}{\sum _{s=1}^S w_i^s}\right) . \end{aligned}$$

where *n* is the number of data points, $\theta ^s$ are draws from the full posterior $p(\theta |y)$, $s=1,\dots ,S$ represent the *S* draws, and $w_i^s$ is a vector of weights that are the Pareto Smoothed importance ratios built through an algorithm described in the LOO original paper^56^. The best model is associated with the smallest LOO value.

## Supplementary information

Supplementary information.

## Data Availability

We are pleased to make available the source-code and datasets accompanying this research. The projects files are available at https://github.com/denadai2/bayesian-crime-multiple-cities/.
