# Supplementary material for: Socio-economic, built environment, and mobility conditions associated with crime: a study of multiple cities
Source: Sci Rep. 2020 Aug 17;10:13871. doi: 10.1038/s41598-020-70808-2 (PMC7431538; doi:10.1038/s41598-020-70808-2)
Supplement: Supplementary file 1 — Supplementary information. [file 41598_2020_70808_MOESM1_ESM.pdf]

Supplementary Information
Socio-economic, built environment, and mobility conditions associated with crime: A study of multiple cities

Marco De Nadai et al.

Contents

Supplementary Note 1 Walkability **2**

Supplementary Note 2 Crime measure **3**

Supplementary Note 3 Data sources **4**
Supplementary Note 3.1 Evaluation of TimeGeo traces 5

Supplementary Note 4 The spatial model **16**

Supplementary Note 5 Alternative spatial models **20**

Supplementary Note 6 Alternative Connectivity Matrices **22**

Supplementary Note 7 Spatial model decomposition **27**

Supplementary Note 8 Improvement analysis **30**

Supplementary Note 9 Auto-correlation of features **34**

Supplementary Note 10 The minimal model **34**

Supplementary Note 11 Corehood tests **35**

Supplementary Note 12 Disentangled crime types **38**

Supplementary Note 1 Walkability

We determine the *walkability* of a neighbourhood through its accessibility to the nearest Point Of Interests (*e.g.*, convenience stores, restaurants, sport facilities). The concept of *walkability* is empirically calculated in many different ways. However, one of the most accepted one is Walk Score [11]. We here describe and compute the *walkability* score for our cities consistently with the methodology proposed by [11], as Walk Score is not available for all the cities we consider.

Thus, for each city block b we first collect an ordered list of n_c closest Point Of Interests (POIs) belonging to category c:

$$\text{closest}(b, c) = [p_1, p_2, \ldots, p_{n_c}] \tag{S1}$$

where p_1 is the closest POI of category c to b, p_2 is the second closest and so on so forth. And then we compute the *walkability* score as:

$$\text{walk}_i = \sum_{c \in C} \sum_{i}^{n_c} w_{c,i} \, \text{distance}(b, \text{closest}(b, c)_i) \tag{S2}$$

where C is the set of categories (i.e. Food, Shops, Grocery, Schools, Entertainment, Parks and outside, Coffee, Banks, Books), distance is the street-network distance decay function (explained later), and $w_{c,i}$ is a weighting factor that depends on both the category c and the i-est closest POI.

In categories where depth of choice is important, multiple POIs are considered (i.e. $n_c > 1$). For example, restaurants and bars are combined in a single category due to their overlapping function. They are the most frequent walking destination, hence we include 10 counts of places to account for the depth of offer in the neighbourhood. The shopping category represent all the retails where people can buy products such as clothes, gifts, etc. They are common walking destinations and they are commonly described as important for the attractiveness of a place. Thus, we considered 5 counts of places for this category. Coffee shops are also important for the neighbourhood, but not as important as restaurants and shopping places. Thus, we considered 2 counts for this category. For other categories only the distance from the nearest POI is calculated. These parameters are consistent with Walk Score [11]. The definitions of w and n_c as summarized in Table S1.

The amenities are extracted from Foursquare, a crowd-sourced project where people participate in an online game where they check-in places where they go.

The distance decay function computes an importance weight to each POI reachable from a starting point. Similarly to Walk Score, we use a polynomial distance that assigns the maximum score to amenities $\sim$ 500 meters far from the starting point, then the score decays quickly until 1500 meters, where it first slows down then it goes to zero. The distance is along the street network, instead of the geometric distance (see Figure S1).

Category	n_c	w
Grocery	1	[3]
Food	10	[.75, .45, .25, .25, .225, .225, .225, .225, .2, .2]
Shops	5	[.5, .45, .4, .35, .3]
Schools	1	[1]
Entertainment	1	[1]
Parks and outside	1	[1]
Coffee	2	[1.25, .75]
Banks	1	[1]
Books	1	[1]

Table S1: Additional details to compute the *walkability* score. n_c is the number of retrieved Point of Interests, while w is an ordered sequence of weights applied to the distances to the nearest n_c Point of Interests.

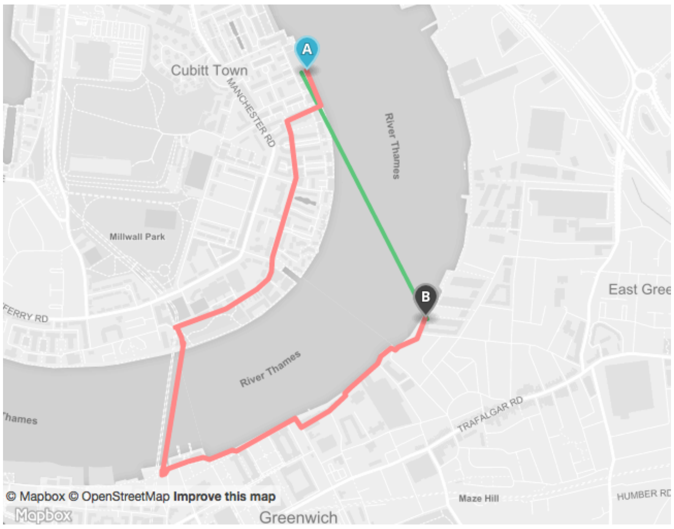

Figure S1: The geometric distance (green) and the shortest path walking distance (red) between two points.

Supplementary Note 2 Crime measure

In criminology, there are usually two ways to assess crime: crime counts and crime rates. The main difference between the two consists on how the population at risk is modelled. The former assumes that the importance of the population at risk has to be found by the model, while the latter assumes that importance is equal to one.

Usually, the population at risk is defined as the residential population. Recently, some scholars have discussed the costs and benefits of alternative denominators such as the ambient population [19]. Different ways exist on how to compute it, including the use of satellite imagery [19], census data [60] and Foursquare check-ins [53].

However, it is not clear whether using residential rates, ambient rates, nor the policy implications of using one or the other. Moreover, the bias of ambient rates can be

potentially lead to misleading interpretation of crime. In this research, we thus prefer to describe crime counts controlling for residential and ambient population. Thus, it is also possible to describe their relative role through the β coefficients of the regression.

Supplementary Note 3 Data sources

In Table S2 we list all the sources and data availability for the information used in this paper. We note that most of the datasets are released via web links without any guarantee that links do not break when the original website gets updated. Our aggregated datasets are instead released with a permanent Digital Object Identifier.

Here we briefly describe each data source we used:

American Community Survey (ACS)[1] [17] is an ongoing survey by the U.S. Census Bureau containing information about the population, employment, ethnicity and income for various spatial aggregation levels. We used this survey for the poverty, population, employment, ethnicity mix and block groups boundaries for the U.S. cities.

TIGER/Line Shapefiles [18] is a dataset released by the U.S. Census Bureau containing the spatial data for the census. We used it to extract the unused areas and the blocks for U.S. cities.

Mapa de referencia (IDECA) [12] is the reference map for Bogotá. It contains various spatial informations such as the blocks (Manzana) and the blocks groups (Barrios), but also the land use information, buildings, unused areas, boundaries of Bogotá.

Sisbén in the Identification System III is the Colombian project to track and help deprivated people. It collects the number of people require special economical needs. We used it to compute one of the variables of disadvantage in Bogotá.

BostonGIS Boston boundary [5] contains the boundary of Boston.

Los Angeles Neighborhood council boundaries [13] are the Official Certified Neighborhood Council boundaries in the City of Los Angeles created and maintained by the Bureau of Engineering/GIS Mapping Division. We aggregated them to form the boundary of Los Angeles.

City of Boston building outlines [2] is the official planimetric dataset created initially from a flyover in 2011 and updated daily based on address requests and permit data. We used it for the buildings of Boston. It is composed by a spatial dataset outlinining the buildings humanly annotated from satellite imagery and aerial data.

Building footprints in Chicago [7] is the official building footprints data source in Chicago. It is composed by a spatial dataset outlining the buildings humanly annotated from satellite imagery and aerial data.

U.S. Crime incidents [15, 3, 8] extracted from the geo-located incident reports provided by Boston Police Department, Chicago Police Department's CLEAR (Citizen Law Enforcement Analysis and Reporting), and Los Angeles Police Department (LAPD).

[1] `https://www.census.gov/programs-surveys/acs`

Bogotá Crime incidents is released by the Ministerio de Defensa Nacional - Direccion de Investigacion Criminal and Interpol. It contains geo-located incidents for all the crimes in Bogotá. Researchers should contact them and sign the Non-Disclousure Agreement (NDA) to access the micro-data for privacy reasons.

Parcels [4, 9, 16]. We used the property tax assessment for the counties containing the analyzed US cities to determine the land use of each property, and thus block. As Chicago's parcels land use are often unavailable, we also used the CMAP Land Use Inventory [10], which is updated every five years and created for land use and transportation research. We associated the land use of CMAP to the parcels of Chicago.

Censo General de Población 2005 is the Colombian national census. It provides micro-data at the block level for the residential stability, population, poverty, employment, ethnicity of Bogotá. We had access to the micro-data through the Departamento Administrativo Nacional de Estadistica (DANE). Researchers should contact them and sign the Non-Disclousure Agreement (NDA) to access the micro-data for privacy reasons.

OSM[2]. OpenStreetMap is a community driven project to map the world. It allows users to edit maps, insert objects and it releases the data under the ODbL license. We used it to extract the street network used to compute the walkability.

Mobile phone data were privately released to the authors. This data consists of the mobile phone activity logs of all mobile phone users across a specific carrier in each city under investigation. Activity logs include received and made calls, SMS activity, and various location signals may also be included.

The data is about 1.92, 1.5, and 3.2 million anonymous mobile phone users for the Greater Boston area, Bogotá and Los Angeles respectively. Since mobile phone data inherently contains noise such as discontinuities that do not represent actual human mobility, we apply a procedure generally used for GPS traces, the so-called stay point algorithm [51, 32, 85]. Specifically, we extract the stop locations of people by classifying all places in *pass-by* and *stay* points with a temporal threshold of 10 minutes and 300 meters. Then, we apply the Timegeo model [52] to fit and simulate reliable geo-located mobility. More details are presented in the following section.

Supplementary Note 3.1 Evaluation of TimeGeo traces

We follow the procedure described in Jiang *et al.* [52] to fit and carefully evaluate our model in Boston, Bogotá and Los Angeles.

The full description of the TimeGeo model is not the focus of this research. However, we briefly describe how the generated traces are validated with the travel surveys of each city. We refer the interested reader to the original paper [52] and its extensively described procedure in the supplementary.

We validated TimeGeo traces with the National Household Travel Survey (NHTS) [82], Bogotá Survey of Mobility [31] and California Household Travel Survey (CHTS) [26]. To do so, we have to: 1) validate how representative they are with the entire population; 2)

[2] `http://www.openstreetmap.org`

validate how well generated traces describe individual mobility; and 3) how well they describe human behaviour. Finally, they are compared with the aggregated Origin-Destination (OD) matrices between spatial areas, showing a high degree of correlation.

Thus, we begin by comparing TimeGeo traces with the real population. CDRs data are not coming from 100% of the population. On the contrary, they largely depend on the market share of the mobile operator. Thus, we first have to expand the millions of users we analyze to the entire population of the city (e.g. Boston). An expansion factor is calculated for each tract as the ratio of the census population and the number of residents identified in the CDR data set. For each census tract with less than 10 CDR residents (around 10 in the study area), the expansion factor is set to 0 to ensure that we do not overweight users that are not representative for a given census tract. Figure S2 shows the validation of Boston, Los Angeles and Bogota before and after the validation. The data is valid and representative of the entire population.

Then, we validate individual mobility traces. Modelling individual behaviour is considered very challenging due to the bursty and heterogeneous characteristics of human behaviour [32, 24]. Figure S3 shows how well TimeGeo generated traces describe human behaviour in two of the three cities. We show that the stay duration, number of visited places and travel distance are well described by TimeGeo. We note that we do not expect that the divergence of stay duration in Boston affects our result, especially for the attractiveness (calculated for stops of 1 hour).

Finally, we compare how well TimeGeo describes the different purposes of trips. The literature of transportation research usually defines three types of trips, namely home-based work (HBW), non-home based (NHB) and home-based other (HBO) [35, 81, 29, 26, 82, 34, 61]. A HBW trip happens if a user is observed to travel between home and work, a NHB trip is counted if a user moves between two non-home stay points, while a HBO trip is counted if a user is observed moving between their home location and a location labelled as other [81]. Figure S4 shows that TimeGeo well describes the purposes of the trips and departure areas in the city.

Descriptive statistics

Figure S5 shows that crime is not evenly distributed in space and crime distribution in the cores. Figure S6 instead shows the different distribution of crime types across cities. It is strikingly how different is the distribution of crimes especially for criminal homicides, burglary, and motor vehicle theft. For instance, Los Angeles has the highest number of motor vehicle thefts.

Figure S7 shows the distribution of the main social disorganization features and of the walkability feature. We observe that the distributions of disadvantage and ethnic diversity composite indexes are quite different for Bogotá. The former is very heterogeneous in Bogotá, while the others are less heterogeneous. The latter shows that Bogotá has very low ethnic diversity compared to US cities. This might also be related to the definition of the different ethnic groups in the census. As expected, walkability is very low in some parts of Bogotá.

Figure S8 shows the distribution of the main built environment features. From this

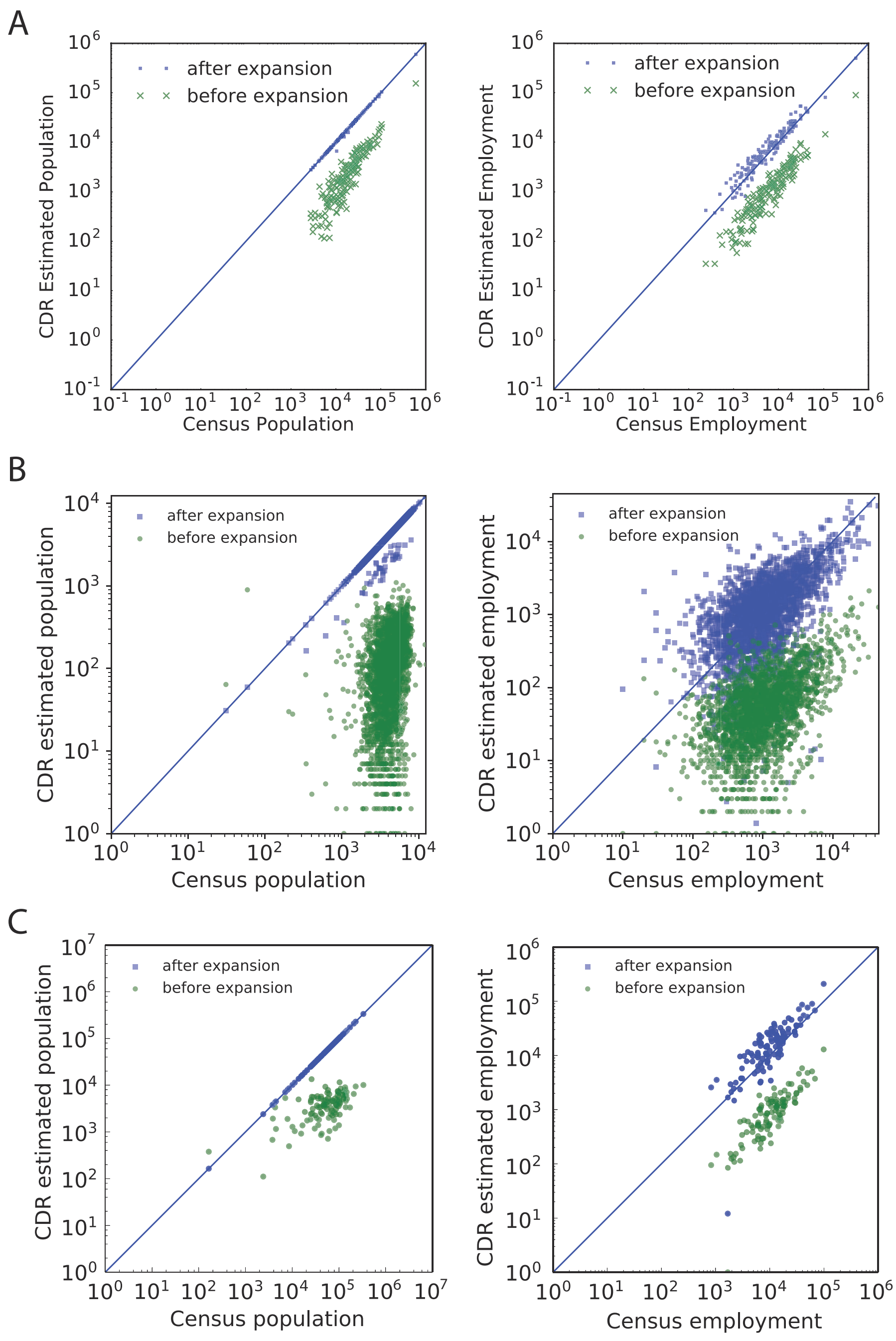

Figure S2: Validation of the home and work labeling for the users with detected home versus the census residential population in each city. A (Boston), B (LA), C (Bogota).

figure, it emerges that the built environment is very diverse across cities. Particularly, the

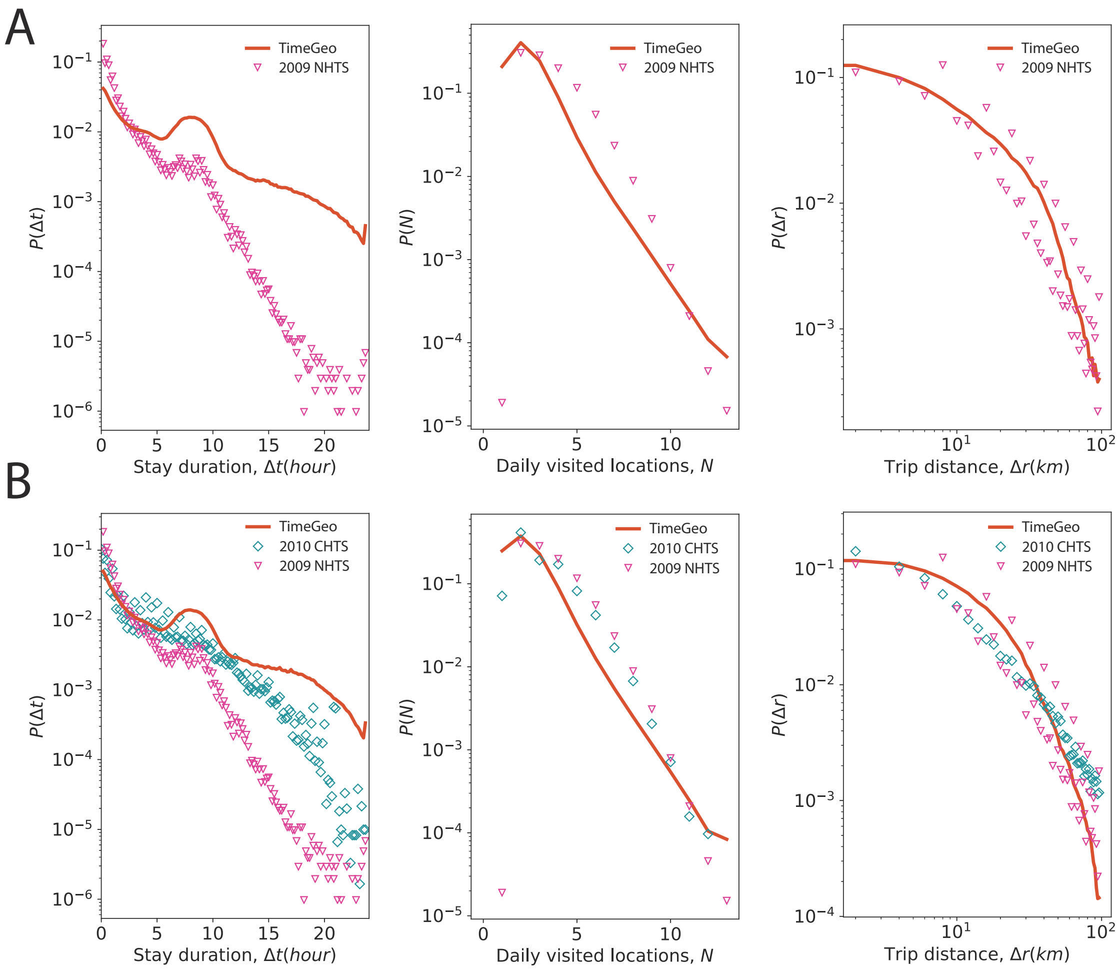

Figure S3: Distribution comparisons of individual mobility between survey data and TimeGeo generated traces. A (Boston), B (LA).

Jane Jacobs' definition of small blocks is surprisingly different even between US cities.

Figure S9 shows the distribution of the main core features. We observe high similarity between Boston and Chicago for Food, Nightlife and Shops POIs. Nightlife is comparable between Los Angeles and Bogotá. The population is much more diversified in Bogotá's cores.

Figure S10 shows the distribution of ambient population and attractiveness. Boston has corehoods that are much more attractive than other cities.

Figure S11 shows the distribution of sizes of cores. We can see that cores in Bogotá and Los Angeles are slightly bigger than those in Chicago and Boston.

**Type**	**City**	**Open Data**	**Data is shared**	
			Raw	Aggregated
Blocks	Bogotá		✓	✓
	Boston	✓	✓	✓
	Chicago	✓	✓	✓
	LA	✓	✓	✓
Census Blocks	Bogotá	✓[12]	✓	✓
	Boston	✓[18]	✓	✓
	Chicago	✓[18]	✓	✓
	LA	✓[18]	✓	✓
Boundaries	Bogotá		✓	✓
	Boston	✓[5]	✓	✓
	Chicago	✓[6]	✓	✓
	LA	✓[13]	✓	✓
Buildings	Bogotá	✓[1]	✓	✓
	Boston	✓[2]	✓	✓
	Chicago	✓[7]	✓	✓
	LA	✓[14]	✓	✓
Crime	Bogotá			✓
	Boston	✓[3]	✓	✓
	Chicago	✓[8]	✓	✓
	LA	✓[15]	✓	✓
Employment and Ethnic mix	Bogotá			✓
	Boston	✓[17]	✓	✓
	Chicago	✓[17]	✓	✓
	LA	✓[17]	✓	✓
Land Use	Bogotá	✓[12]	✓	✓
	Boston	✓[4]	✓	✓
	Chicago	✓[9, 10]	✓	✓
	LA	✓[16]	✓	✓
Mobile phone data	All but Chicago			✓
POIs	All			✓
Population	Bogotá			✓
	Boston	✓[17]	✓	✓
	Chicago	✓[17]	✓	✓
	LA	✓[17]	✓	✓
Poverty	Bogotá			✓
	Boston	✓[17]	✓	✓
	Chicago	✓[17]	✓	✓
	LA	✓[17]	✓	✓
Residential stability	Bogotá			✓
	Boston	✓[17]	✓	✓
	Chicago	✓[17]	✓	✓
	LA	✓[17]	✓	✓
Street network	All	✓	✓	✓

Table S2: Description of the data sources to replicate the paper. Most of the data is shared along this paper in raw format, while a subset of it (POIs and mobile phone data) could be shared only in an aggregated format for license and privacy reasons.

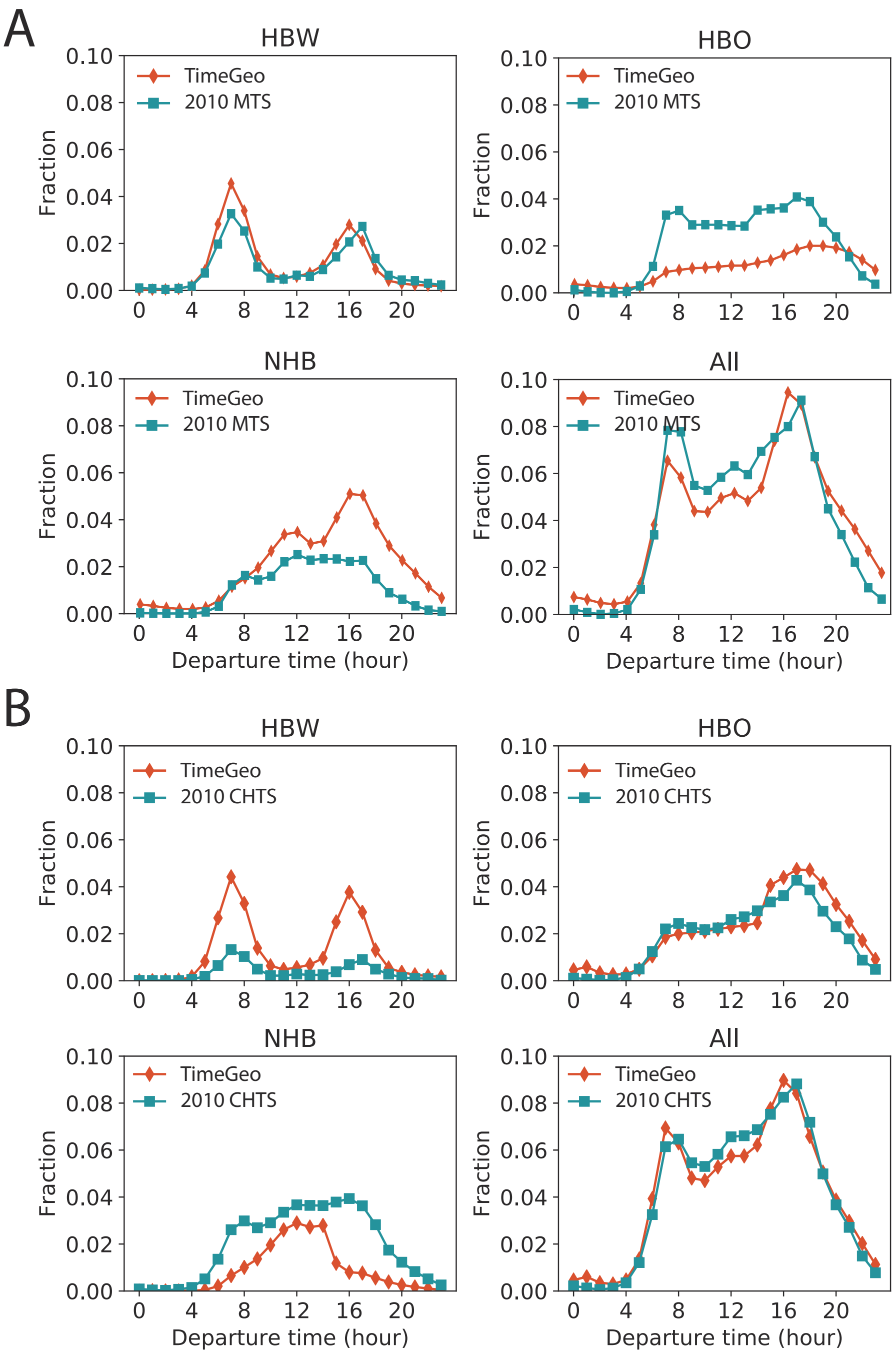

Figure S4: Distribution comparisons of individual mobility by trip type between survey data and TimeGeo generated traces. A (Boston), B (LA), shows the hourly volume by trip purpose.

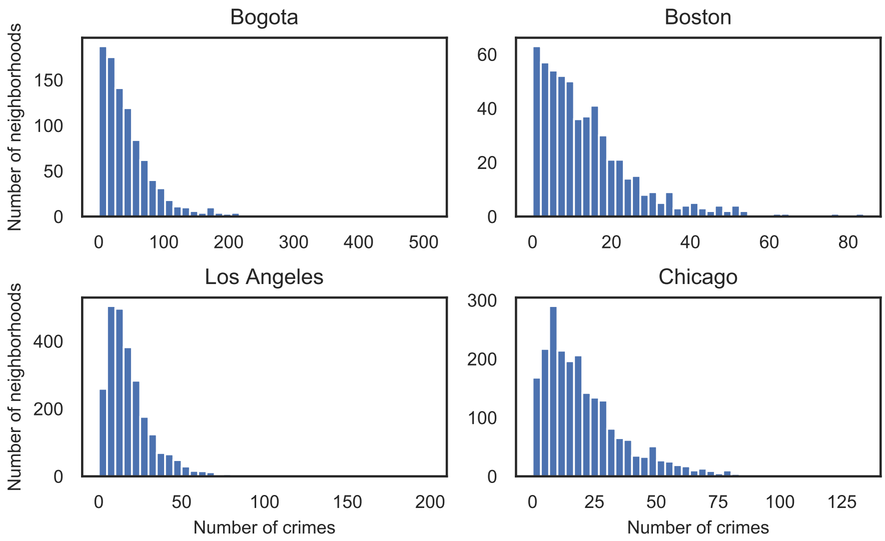

Figure S5: Distribution of the number of total committed crimes for each neighborhood.

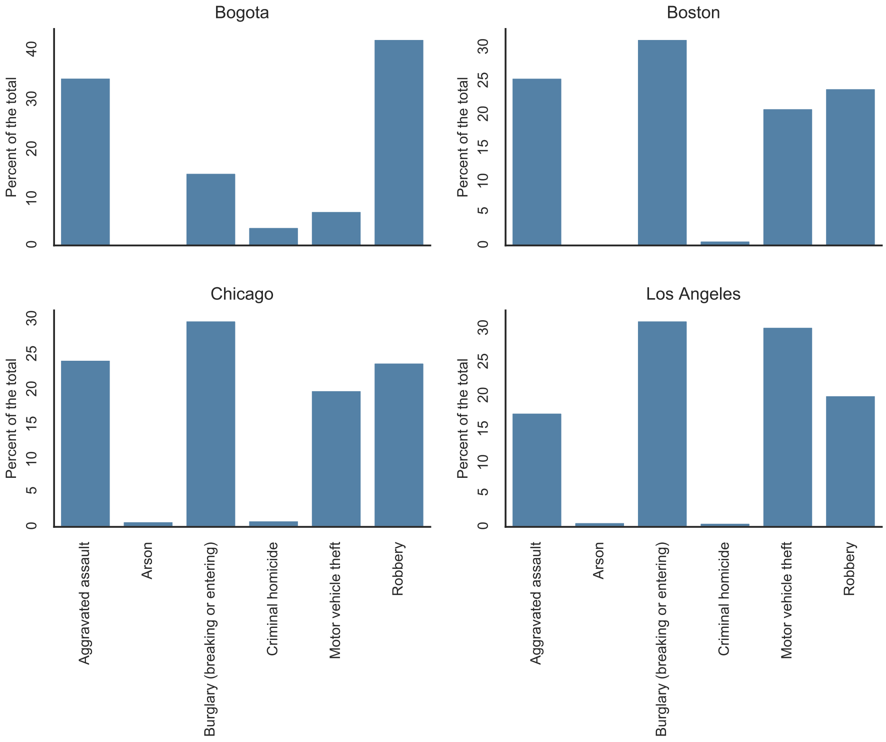

Figure S6: Distribution of the number of committed crimes per type of crimes.

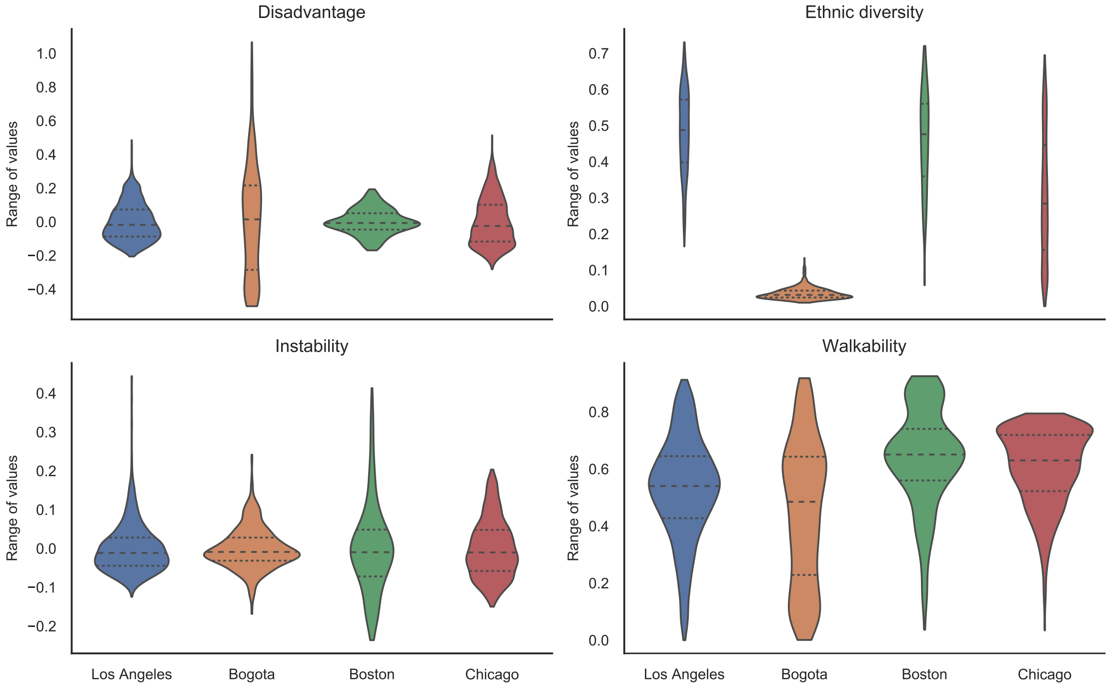

Figure S7: Distribution of some of the features (i.e. disadvantage, ethnic diversity, instability, walkability) of the corehood.

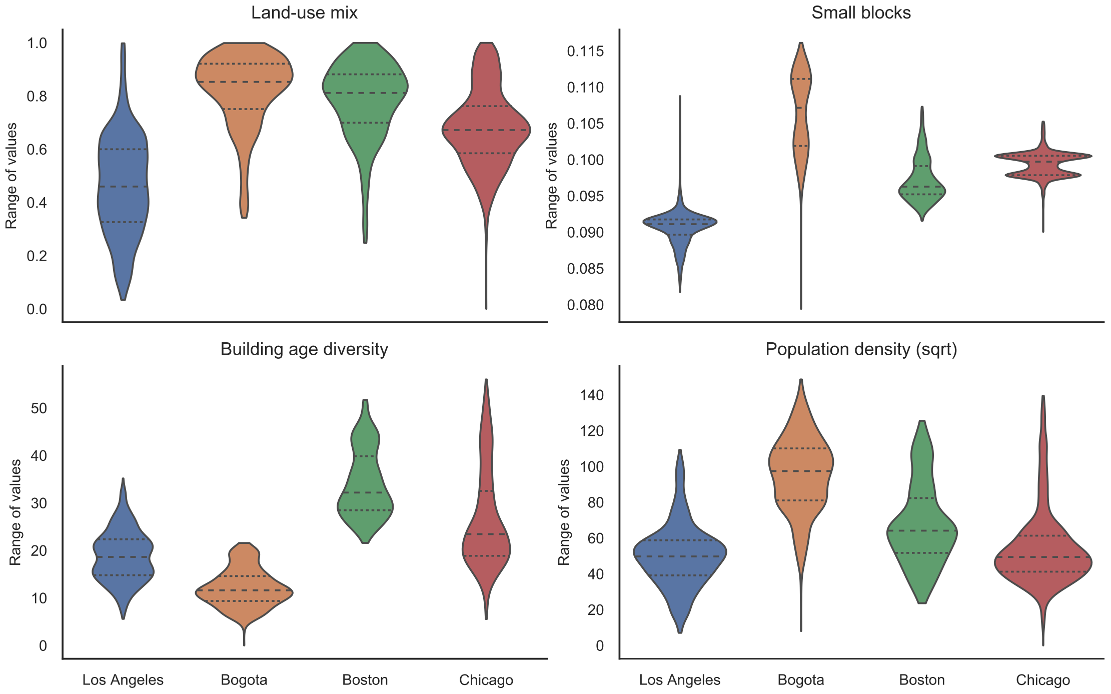

Figure S8: Distribution of some of the features (i.e. land use mix, small block, building age diversity, population density) of the corehood.

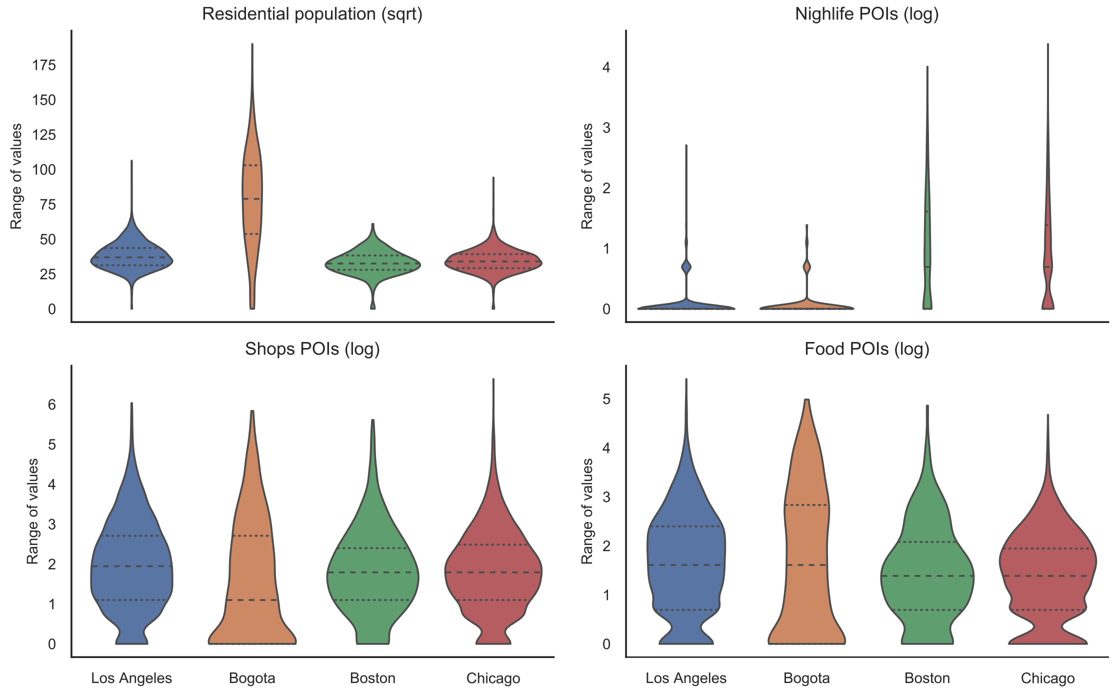

Figure S9: Distribution of some of the features (i.e. residential population, nightlife POIs, shops' POIs, food POIs) of the core.

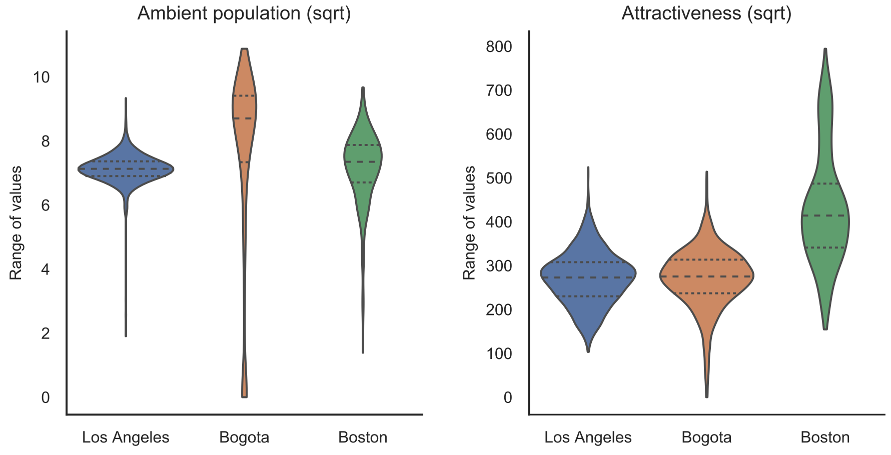

Figure S10: Distribution of the features extracted from mobile phone data (i.e. ambient population, attractiveness) for the corehood. Chicago does not have mobile phone data.

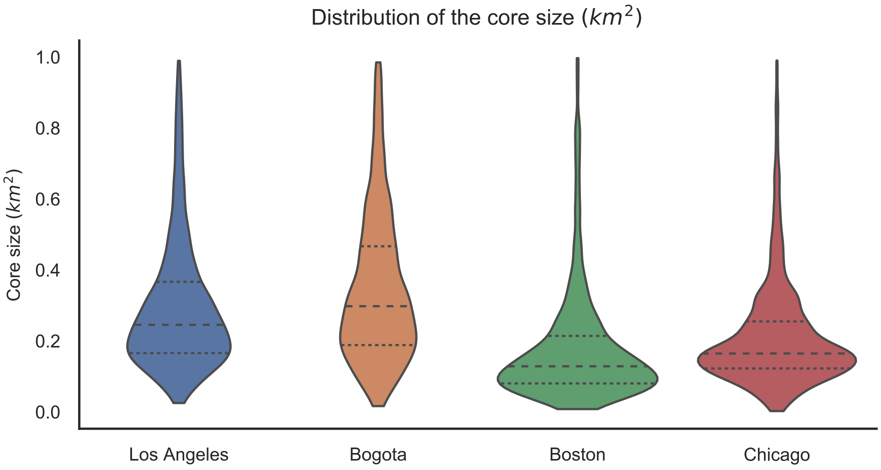

Figure S11: Distribution of all the features of the core. Cores in Bogotá and Los Angeles are slightly bigger than those in Chicago and Boston.

Supplementary Note 4 The spatial model

Crime patterns have been observed to be highly concentrated in the space, overdispersed [67], and positively spatial auto-correlated. The spatial auto-correation of a variable y is usually computed through the Moran's I coefficient [62] $\text{MC}(y)$:

$$\text{MC}(y) = \frac{N}{\mathbf{1}'\mathbf{C}\mathbf{1}} \frac{\mathbf{y}'\mathbf{MCMy}}{\mathbf{y}'\mathbf{My}} \tag{S3}$$

where $\mathbf{1}$ is a $N \times 1$ vector of ones, $\mathbf{y}$ is a $N \times 1$ vector of variable values, $\mathbf{C}$ is a $N \times N$ symmetric connectivity matrix whose diagonal elements are zero, and $\mathbf{M}$ is a $N \times N$ projection matrix. The projection matrix can be either $\mathbf{M} = \mathbf{I} - \mathbf{1}\mathbf{1}'/N$ or $\mathbf{M} = \mathbf{I} - \mathbf{X}(\mathbf{X}'\mathbf{X}) - \mathbf{X}'$ to approximate the spatial lag and spatial error models respectively.

When $\text{MC}(y) > 0$ there is positive auto-correlation, negative otherwise. In the former case, places with high crime tend to be near places with high crime, in the latter places with high crime are near places with low crime. When MC is not near zero, regression models might exhibit spatial correlation in the residuals, thus invalidating the assumption of independence of the errors. In these cases, regression models should account for the spatial auto-correlation between spatial units.

In our paper, we model the crime counts in a city with a Negative Binomial (NB) regression, and we account for the spatial auto-correlation with the Bayesian Spatial Filtering (BSF) [49] approach. The NB model is defined as:

$$\text{NegBinomial2}(y \mid \mu, \phi) = \binom{y + \phi - 1}{y} \left(\frac{\mu}{\mu + \phi}\right)^{y} \left(\frac{\phi}{\mu + \phi}\right)^{\phi} \tag{S4}$$

where the mean and variance of y are: $\mathbb{E}[Y] = \mu$ and $\text{Var}[Y] = \mu + \frac{\mu^2}{\phi}$. We use the logarithm as link function for the NB and $\phi^{-1} \sim N(0, 1)$.

The BSF model is defined as:

$$\log(\mathbb{E}[Y]) = \beta_0 + \boldsymbol{X}\beta + \mathbf{E}\gamma \tag{S5}$$

$$\gamma|\rho \sim \mathcal{N}(\mathbf{0}, (\rho\mathbf{E}^{\intercal}\mathbf{QE})^{-1}) \tag{S6}$$

$$\rho \sim \Gamma(0.5, 2000). \tag{S7}$$

where $\mathbf{Q}$ is the Laplacian of $\mathbf{C}$, $\mathbf{E}$ is the matrix composed by the eigenvector extracted from the $\mathbf{MCM}$ in Equation (S3), and ρ is a Gamma with a large mean to discourage artifactual spatial structures in the posterior [21, 49]. $\mathbf{C}$ can be defined in different ways but the main model uses a binary matrix representing the pairwise relation between areas. In Section Supplementary Note 6 we analysed other specifications of $\mathbf{C}$.

Since we want to account for the correlation between the features, and well generalize the model, we apply a Ridge penalty to the β coefficients and the QR decomposition to decorrelate covariates and, thus, the resulting posterior distribution. Thus, we model β_0 and β as:

$$\begin{aligned} \beta_0 &\sim \mathcal{N}(0, 1) \\ \beta|\tau &\sim \mathcal{N}(0, \tau) \\ \tau &\sim \mathcal{C}^{+}(0, 1). \end{aligned}$$

The alternative formulation that does not account for the auto-correlation is a NB model with Ridge penalty on the beta coefficients, which is defined as:

$$\log(\mathbb{E}[Y]) = \beta_0 + \boldsymbol{X}\beta$$

where $C^+(0,1)$ is the half-Cauchy distribution with a mean of zero and a scale parameter of one. We chose the half-Cauchy as suggested by Andrew Gelman's work [38].

In Table S3 we show the NB model exhibits a strong positive spatial auto-correlation in the residuals, while the BSF model does not, as expected. The models based on BSF are also superior in the LOO and R^2_c.

Test for overdispersion

The NB model is motivated by the extra-Poisson variability of the crime distribution in the city. We can test the need of overdispersion through the Potthoff-Whittinghill and the Lagrange multiplier test.

The Potthoff-Whittinghill index of dispersion test [70] rejects the hypothesis of no overdispersion. It is defined as:

$$\sum_{i=1}^{N}(y_i - \bar{y})^2/\bar{y} \tag{S8}$$

which is approximately a chi-square distribution with $k-1$ degrees of freedom. We also apply the Lagrange multiplier test, defined as:

$$\frac{(\sum_{i=1}^{N}\mu_i^2 - n\bar{y})^2}{2\sum_{i=1}^{N}\mu_i^2} \tag{S9}$$

With one degree of freedom, the test appears to be significant – the hypothesis of no overdispersion is again rejected.

Selection of E eigenvectors

Following seminal literature of eigen-based spatial modelling and filtering [78], we select the first k eigenvectors from $\mathbf{E}_{full} = \mathbf{MCM}$, where $\mathbf{C}$ is a spatial matrix that describes the graph between spatial locations, while $\mathbf{M} = \mathbf{I} - \mathbf{X}(\mathbf{X}'\mathbf{X}) - \mathbf{X}'$, which is an approximation of the spatial error model.

The associated sets of eigenvalues $(\lambda_1, \lambda_2, \ldots, \lambda_N)$ from the $\mathbf{MCM}$ decomposition assess the strength of a spatial pattern. A vector i with $\lambda_i > 0$ describes positive spatial auto-correlation, while vectors with $\lambda_i < 0$ describe negative spatial auto-correlation. Spatial models are notoriously inefficient at dealing with negative spatial auto-correlation, which is also rather rare. Thus, consistently with literature [28] we focus on positive auto-correlation and we select those vectors having $\lambda' = \lambda/\lambda_{max} >= 0.25$, where λ_{max} is the maximum value among the eigenvalues.

Test for residual auto-correlation

We test for the presence of auto-correlation in the models' residuals to the Moran's I. However, since our model is not an Ordinary Least Squares, we used in our tables a corrected version of Moran's I that is specifically tailored for log-linear relationships [58]. The index is defined as:

$$MC_p(r) = \frac{N}{\sum_{i,j=0}^{N} \mathbf{C}_{i,j}} \frac{r'\mathbf{C}r}{r'r} \tag{S10}$$

where N is the number of spatial units, and r is the vector of residual errors.

We did not find significant residual spatial auto-correlation in our spatial models.

Additional results with the DIC

Recent literature on Bayesian models evaluation strongly discourage the use of the Deviance Information Criterion (DIC) [76] due to its numerous disadvantages [77, 83]. However, DIC has been used extensively for practical model comparison in many disciplines as it is often available in many software packages like WinBUGs and INLA. Thus, we also show in this Supplementary Information the DIC performance for all the methods. DIC may be parametrized in different ways. In this manuscript, we follow Gelman *et al* [37] defining DIC as:

$$\text{DIC} = \bar{D} + \frac{1}{2} v\hat{a}r(D)$$

where $\bar{D} = -\frac{2}{G}\sum_{g=1}^{G} \log p(y|\theta_g)$ is usually called average deviance, G is the number of posterior samples of θ, and $v\hat{a}r(D) = \frac{1}{G-1}\sum_{g=1}^{G}(D(\theta^g) - \bar{D})^2$ measures the model complexity, and $D(\theta^g) = -2\log p(y|\theta_g)$.

It is worth highlighting that our results hold also for the DIC metric.

Model	BSF				NB			
	LOO	R_c^2	DIC	MC_p	LOO	R_c^2	DIC	MC_p
**Bogotá**								
Core	**-3897**	**0.75**	**7844**	**-0.034**	-4126	0.53	8250	0.454
Social-disorganization (SD)	**-3891**	**0.75**	**7829**	**-0.042**	-4079	0.58	8154	0.355
Built environment (BE)	**-3881**	**0.76**	**7803**	**-0.036**	-4061	0.61	8118	0.371
Mobility (M)	**-3804**	**0.80**	**7649**	**-0.042**	-4034	0.64	8066	0.461
SD+BE	**-3880**	**0.76**	**7813**	**-0.035**	-4013	0.65	8022	0.286
SD+M	**-3794**	**0.81**	**7636**	**-0.049**	-3988	0.67	7973	0.373
BE+M	**-3819**	**0.80**	**7680**	**-0.026**	-3980	0.68	7957	0.362
SD+BE+M (Full)	**-3809**	**0.80**	**7671**	**-0.040**	-3941	0.71	7879	0.284
**Boston**								
Core	**-2035**	**0.65**	**4091**	**-0.005**	-2210	0.22	4419	0.418
Social-disorganization (SD)	**-2019**	**0.68**	**4056**	**-0.004**	-2088	0.55	4175	0.235
Built environment (BE)	**-2015**	**0.68**	**4050**	**-0.034**	-2169	0.37	4336	0.310
Mobility (M)	**-2000**	**0.70**	**4025**	**-0.026**	-2140	0.45	4280	0.351
SD+BE	**-1987**	**0.72**	**4002**	**-0.043**	-2030	0.65	4059	0.108
SD+M	**-1973**	**0.73**	**3978**	**-0.031**	-2011	0.67	4020	0.105
BE+M	**-1990**	**0.72**	**4006**	**-0.033**	-2109	0.52	4217	0.263
SD+BE+M (Full)	**-1957**	**0.75**	**3944**	**-0.039**	-1993	0.70	3985	0.086
**LA**								
Core	**-9665**	**0.68**	**19398**	**0.032**	-10757	0.17	21512	0.647
Social-disorganization (SD)	**-9528**	**0.72**	**19122**	**0.004**	-10042	0.55	20082	0.417
Built environment (BE)	**-9629**	**0.69**	**19320**	**0.005**	-10618	0.27	21234	0.616
Mobility (M)	**-9570**	**0.70**	**19183**	**0.018**	-10658	0.24	21314	0.628
SD+BE	**-9509**	**0.72**	**19068**	**-0.010**	-9989	0.57	19972	0.366
SD+M	**-9467**	**0.73**	**18991**	**-0.002**	-10003	0.57	20002	0.445
BE+M	**-9585**	**0.70**	**19227**	**0.011**	-10570	0.30	21139	0.618
SD+BE+M (Full)	**-9453**	**0.74**	**18957**	**-0.010**	-9967	0.58	19929	0.388
**Chicago**								
Core	**-8415**	**0.68**	**16879**	**0.117**	-9350	0.09	18699	0.542
Social-disorganization (SD)	**-8019**	**0.78**	**16087**	**0.016**	-8390	0.66	16776	0.295
Built environment (BE)	**-8370**	**0.69**	**16808**	**0.093**	-9237	0.21	18474	0.518
SD+BE	**-8002**	**0.79**	**16049**	**0.003**	-8358	0.68	16708	0.283

Table S3: Comparison between the BSF model and the NB model that does not account for the spatial auto-correlation. We evaluate the models through LOO, R_c^2, DIC, and MC_p.

Supplementary Note 5 Alternative spatial models

We tested alternative spatial models that could explain the residual spatial-autocorrelation. Here, we compare the BSF with other two similar, but competitive models: the Random Effects Eigenvector Spatial Filtering (RE-ESF) [63] and the Linear ESF model [78]. The ESF model is defined as:

$$
\begin{aligned}
log(\mathbb{E}[Y]) &= \alpha + \boldsymbol{X}\beta + \mathbf{E}\gamma \\
\gamma|\rho &\sim \mathcal{N}(0, \rho\lambda) \\
\rho^{-2}|\nu &\sim \Gamma(\nu/2, \nu/2) \\
\nu &\sim \Gamma(2, 0.1).
\end{aligned}
$$

where λ is the vector $L \times 1$ of the eigenvalues associated with $\mathbf{E}_{\text{full}}$, and ρ is chosen to constrain the spatial random effects γ and to avoid that they penalize too much the fixed effects. To ensure limited variance, v is limited to an upper value of 2.

The RE-ESF instead assumes γ to be random such that:

$$
\begin{aligned}
\gamma|\alpha, \rho &\sim \mathcal{N}(0, \rho\mathbf{\Lambda}(\omega)) \\
\omega^{-1} &\sim \Gamma(2, 5).
\end{aligned}
$$

where $\lambda(\omega) = \frac{\sum_l \lambda_l}{\sum_l \lambda_l^{\omega}} \lambda_l^{\omega}$ is a multiplier that represents the scale of spatial variance, and ω is a parameter to be found.

Table S4 shows that no model clearly outperforms another, suggesting that they are almost equivalent in a Full Bayesian setting.

Model	BSF			RE-ESF			ESF		
	LOO	DIC	MC_p	LOO	DIC	MC_p	LOO	DIC	MC_p
**Bogotá**									
Core	**-3896**	7841	-0.034	-3899	**7835**	-0.045	-3902	7841	-0.041
Social-disorganization (SD)	**-3891**	7833	-0.043	-3894	7834	-0.052	-3896	**7826**	-0.049
Built environment (BE)	**-3881**	7807	-0.036	-3882	7808	-0.045	-3883	**7802**	-0.042
Mobility (M)	-3803	7647	-0.042	**-3802**	**7638**	-0.048	-3807	7641	-0.047
SD+BE	**-3881**	**7812**	-0.035	-3883	7814	-0.043	-3884	7812	-0.041
SD+M	**-3795**	7634	-0.050	-3796	**7634**	-0.057	-3798	7634	-0.055
BE+M	-3819	7680	-0.025	**-3817**	7680	-0.033	-3821	**7676**	-0.033
SD+BE+M (Full)	**-3809**	7670	-0.041	-3810	7663	-0.049	-3810	**7656**	-0.046
**Boston**									
Core	-2035	4091	-0.005	**-2034**	4078	-0.016	-2035	**4076**	-0.014
Social-disorganization (SD)	**-2019**	4057	-0.004	**-2019**	4053	-0.018	-2020	**4052**	-0.015
Built environment (BE)	-2015	4050	-0.034	-2014	4034	-0.044	**-2013**	**4031**	-0.044
Mobility (M)	-2000	4025	-0.026	**-1999**	**4006**	-0.035	**-1999**	4006	-0.035
SD+BE	-1987	4002	-0.043	-1987	3990	-0.057	**-1986**	**3984**	-0.057
SD+M	-1973	3978	-0.031	-1972	3967	-0.046	**-1971**	**3958**	-0.045
BE+M	-1990	4006	-0.033	**-1988**	3986	-0.043	**-1988**	**3986**	-0.042
SD+BE+M (Full)	-1957	3944	-0.039	-1957	3934	-0.054	**-1956**	**3927**	-0.053
**LA**									
Core	-9665	**19398**	0.032	**-9663**	19409	0.028	-9672	19422	0.028
Social-disorganization (SD)	**-9528**	19122	0.004	-9530	19119	-0.001	-9534	**19116**	-0.001
Built environment (BE)	-9629	19320	0.005	**-9628**	**19310**	0.002	-9638	19316	0.001
Mobility (M)	-9570	**19183**	0.018	**-9569**	19202	0.014	-9576	19199	0.014
SD+BE	**-9509**	**19068**	-0.010	-9510	19076	-0.014	-9513	19070	-0.014
SD+M	**-9467**	18991	-0.002	-9468	**18990**	-0.006	-9472	18992	-0.006
BE+M	-9585	19227	0.011	**-9584**	**19220**	0.008	-9591	19226	0.007
SD+BE+M (Full)	**-9453**	18957	-0.010	-9455	18970	-0.015	-9458	**18951**	-0.015
**Chicago**									
Core	-8415	16879	0.117	**-8413**	16864	0.114	-8414	**16853**	0.115
Social-disorganization (SD)	**-8019**	16087	0.016	**-8019**	16064	0.012	**-8019**	**16061**	0.013
Built environment (BE)	-8370	16808	0.093	**-8368**	**16792**	0.090	-8370	16799	0.090
SD+BE	**-8002**	16049	0.003	-8003	16039	-0.001	-8005	**16038**	-0.000

Table S4: Results of alternative spatial models applied in each city. No state of the art model in eigenvector spatial filtering emerge as the best model from our test. We evaluate the models through LOO, DIC, and MC_p.

Supplementary Note 6 Alternative Connectivity Matrices

Incorporating spatial relationship in spatial models requires the definition of a connectivity matrix **C** that describes the relationship (if any) between one spatial unit and all the others. One of the most common connectivity matrix is a binary relationship between spatial units, also called topology representation [45], which usually results in a sparse matrix:

$$\mathbf{C} = (c_{i,j}) = \begin{cases} 1, & \text{if } i \text{ is neighbour of } j \text{ and } i \neq j \\ 0, & \text{otherwise} \end{cases} \tag{S11}$$

An alternative formulation is based on distance. For example, Griffith *et al.* [45] define:

$$\mathbf{C} = (c_{i,j}) = \begin{cases} 0, & \text{if } i = j \\ 0, & \text{if } d > t \\ 1 - (d_{i,j}/4t)^2, & \text{if } d \leq t \end{cases} \tag{S12}$$

where t is chosen as the maximal distance that keeps all the spatial units connected, while $d_{i,j}$ is the (Euclidean) distance between the centroids of unit i and j, and t is the maximal distance of a Minimum Spanning Tree (MST) computed on the distance matrix **D** containing the pairwise Euclidean distances between units.

We also tested for a model that accounts for the human mobility connectivity, extracted from mobile phone data. We conducted this analysis for two main reasons. On the one hand, movements between areas can be interpreted as a distance measure in the mobility space. When a spatial model is used with distance connectivity, the assumption is that an area i is linked to an area j through geographical (Euclidean) distance. However, in cities the time to travel between two areas is influenced by geographical barriers, street connectivity, cars, and public transportation. For example, two areas might be connected by an underground transportation link and might result in a higher connection despite the geographical distance between the two. Mobility is also tightly coupled with distance [23, 39, 55] and economic activity [68], thus we interpreted spatial correlation and connectivity through the human connection between two areas, similarly to the commuting flows used by Patuelli *et al.* [69].

On the other hand, recent literature has shown the correlation between human mobility and criminal activity [20, 19, 27, 25, 59, 53]. For example, Camihna *et al.* [27] showed the superlinear scaling of flows between census areas and crime. Boivin and Felson [25] showed that the number of visiting people in an area is a strong predictor of crime committed by non-residents. Moreover, recent literature highlights the importance of considering connectivity matrices between areas as they could be interpreted as a proxy of spatial mismatch and isolation [42], which was empirically found to be connected with crime [43, 56, 84, 41].

Thus, from TimeGeo traces we aggregated movements into the origin-destination matrix between spatial units and define:

$$\mathbf{C}^* = (c_{i,j}) = \begin{cases} 0, & \text{if } i = j \\ t_{i,j}, & otherwise \end{cases} \tag{S13}$$

where $t_{i,j}$ is the number of trips made, on average, from the unit i to unit j. For the matrix to be comparable with spatial distance, we normalize the flows $t_{i,j}$ by the sum of all the outgoing flows $\sum_j t_{i,j}$ (also called in literature W-scheme). As the matrix $\mathbf{C}$ is not symmetrical:

$$\mathbf{C} = (\mathbf{C}^* + \mathbf{C}^{*\intercal})/2 \tag{S14}$$

As shown in Table S5, the BSF model with contiguity matrix achieves better performance in all the urban settings.

We also tested another normalization for the mobility connectivity matrix, dividing the flows $t_{i,j}$ by the sum of the population of area i and j. This normalization transform the flows in something related to the number of trips per resident between two areas. We observe in Table S6 that the connectivity matrix with W-scheme has better performance.

Human mobility and distance combination

We also tested two different models, named **Mixed1** and **Mixed2**, that use both the distance and the movements of people between sites.

In Mixed1, we based our model on the BSF (see the main paper and Section Supplementary Note 4). However, we used a connectivity matrix $\mathbf{C}$ defined as the mean of the two normalized (in $[0,1]$) matrices defined in Equation (S12) and Equation (S14).

In Mixed2, we instead modeled the distance and the mobility matrices as two separate random effects. Thus, following the aforementioned BSF model:

$$\begin{aligned}
\log(\mathbb{E}[Y]) &= \beta_0 + \boldsymbol{X}\beta + \mathbf{E}_D\gamma + \mathbf{E}_T\omega \\
\gamma|\rho_D &\sim \mathcal{N}(\mathbf{0}, (\rho_D \mathbf{E}_D^\intercal \mathbf{Q}_D \mathbf{E}_D)^{-1}) \\
\omega|\rho_T &\sim \mathcal{N}(\mathbf{0}, (\rho_T \mathbf{E}_T^\intercal \mathbf{Q}_T \mathbf{E}_T)^{-1}) \\
\rho_D &\sim \Gamma(0.5, 2000) \\
\rho_T &\sim \Gamma(0.5, 2000).
\end{aligned}$$

where $\mathbf{Q}_D$ is the Laplacian of Equation (S12), $\mathbf{Q}_T$ is the Laplacian of Equation (S14), while $\mathbf{E}_D$ and $\mathbf{E}_T$ are the selected eigenvectors from the decomposition of Equation (S12) and Equation (S14) respectively.

Table S7 shows the models using the contiguity matrix compared to those using both the distance and mobility matrices. We observe that the model using the contiguity matrix is superior to the Mixed1 and Mixed2 models. We think that this result is due to the auto-correlation of crime events, which might be locally clustered. Thus, the distance and the number of trip between areas are less expressive than the contiguity matrix.

By comparing Table S7 with Table S5 we observe that the use of both distance and mobility is superior to using only the distance or mobility matrices alone just for the Mixed2 model. We think that, while distance and mobility are tied to each other [23, 39, 55], they convey different characteristics to the model, which have to be modelled separately.

Model	Contiguity			Distance			Mobility		
	LOO	DIC	MC_p	LOO	DIC	MC_p	LOO	DIC	MC_p
**Bogotá**									
Core	**-3896**	**7841**	-0.034	-3999	8025	0.016	-4102	8211	0.011
Social-disorganization (SD)	**-3891**	**7833**	-0.043	-3969	7966	0.010	-4037	8077	0.015
Built environment (BE)	**-3881**	**7807**	-0.036	-3971	7979	0.003	-4054	8112	0.012
Mobility (M)	**-3803**	**7647**	-0.042	-3906	7839	0.010	-4008	8019	0.000
SD+BE	**-3881**	**7812**	-0.035	-3949	7927	0.006	-4001	8003	0.019
SD+M	**-3795**	**7634**	-0.050	-3879	7777	0.002	-3943	7886	0.007
BE+M	**-3819**	**7680**	-0.025	-3889	7810	0.002	-3976	7953	0.011
SD+BE+M (Full)	**-3809**	**7670**	-0.041	-3873	7769	0.001	-3928	7856	0.019
**Boston**									
Core	**-2035**	**4091**	-0.005	-2078	4186	0.035	-2193	4389	0.072
Social-disorganization (SD)	**-2019**	**4056**	-0.004	-2037	4101	0.025	-2069	4140	0.022
Built environment (BE)	**-2015**	**4050**	-0.034	-2040	4107	0.010	-2120	4243	0.041
Mobility (M)	**-2000**	**4025**	-0.026	-2013	4053	-0.009	-2127	4253	0.071
SD+BE	**-1987**	**4002**	-0.043	-2007	4035	-0.002	-2030	4060	0.009
SD+M	**-1973**	**3978**	-0.031	-1991	4000	-0.012	-2008	4016	0.001
BE+M	**-1990**	**4006**	-0.033	-2007	4042	-0.007	-2067	4137	0.033
SD+BE+M (Full)	**-1957**	**3944**	-0.039	-1976	3976	-0.008	-1993	3985	0.003
**LA**									
Core	**-9665**	**19398**	0.032	-9966	20023	0.061	-10019	20049	0.026
Social-disorganization (SD)	**-9528**	**19122**	0.004	-9709	19436	0.014	-9899	19798	0.026
Built environment (BE)	**-9629**	**19320**	0.005	-9767	19548	0.017	-9930	19864	0.027
Mobility (M)	**-9570**	**19183**	0.018	-9875	19791	0.050	-9947	19892	0.025
SD+BE	**-9509**	**19068**	-0.010	-9668	19354	0.005	-9914	19827	0.033
SD+M	**-9467**	**18991**	-0.002	-9647	19312	0.016	-9840	19678	0.026
BE+M	**-9585**	**19227**	0.011	-9734	19478	0.021	-9906	19810	0.029
SD+BE+M (Full)	**-9453**	**18957**	-0.010	-9613	19247	0.003	-9886	19766	0.034
**Chicago**									
Core	**-8415**	**16879**	0.117	-8716	17562	0.076			
Social-disorganization (SD)	**-8019**	**16087**	0.016	-8256	16609	0.028			
Built environment (BE)	**-8370**	**16808**	0.093	-8623	17359	0.058			
SD+BE	**-8002**	**16049**	0.003	-8244	16581	0.031			

Table S5: Results of alternative connectivity matrices applied in the spatial model of each city. Chicago does not have mobility information, thus it was not possible to use the mobility matrix. We evaluate the models through LOO, DIC, and MC_p.

Model	Mobility			Mobility - population		
	LOO	DIC	MC_p	LOO	DIC	MC_p
**Bogotá**						
Core	**-4102**	**8211**	0.011	-4126	8250	0.080
Social-disorganization (SD)	**-4037**	**8077**	0.015	-4079	8154	0.074
Built environment (BE)	**-4054**	**8112**	0.012	-4061	8117	0.049
Mobility (M)	**-4008**	**8019**	0.000	-4034	8067	0.044
SD+BE	**-4001**	**8003**	0.019	-4013	8022	0.047
SD+M	**-3943**	**7886**	0.007	-3988	7973	0.048
BE+M	**-3976**	**7953**	0.011	-3980	7957	0.029
SD+BE+M (Full)	**-3928**	**7856**	0.019	-3942	7878	0.034
**Boston**						
Core	**-2193**	**4389**	0.072	-2209	4418	0.121
Social-disorganization (SD)	**-2069**	**4140**	0.022	-2088	4175	0.115
Built environment (BE)	**-2120**	**4243**	0.041	-2169	4337	0.076
Mobility (M)	**-2127**	**4253**	0.071	-2140	4281	0.119
SD+BE	**-2030**	4060	0.009	**-2030**	**4059**	0.022
SD+M	**-2008**	**4016**	0.001	-2011	4020	0.002
BE+M	**-2067**	**4137**	0.033	-2109	4218	0.075
SD+BE+M (Full)	**-1993**	**3985**	0.003	**-1993**	3986	-0.001
**LA**						
Core	**-10019**	**20049**	0.026	-10757	21512	0.197
Social-disorganization (SD)	**-9899**	**19798**	0.026	-10042	20081	0.078
Built environment (BE)	**-9930**	**19864**	0.027	-10618	21233	0.131
Mobility (M)	**-9947**	**19892**	0.025	-10657	21313	0.146
SD+BE	**-9914**	**19827**	0.033	-9989	19973	0.051
SD+M	**-9840**	**19678**	0.026	-10003	20002	0.105
BE+M	**-9906**	**19810**	0.029	-10570	21139	0.124
SD+BE+M (Full)	**-9886**	**19766**	0.034	-9966	19928	0.062

Table S6: Results of alternative normalizations for the mobility matrix. We evaluate the models through LOO, DIC, and MC_p.

Model	Contiguity			Mixed1			Mixed2		
	LOO	DIC	MC_p	LOO	DIC	MC_p	LOO	DIC	MC_p
**Bogotá**									
Core	**-3897**	**7843**	-0.034	-4079	8167	0.052	-3924	7864	0.014
Social-disorganization (SD)	**-3891**	**7828**	-0.042	-4026	8057	0.035	-3913	7844	0.008
Built environment (BE)	**-3881**	7803	-0.036	-4051	8106	0.050	-3886	**7788**	-0.004
Mobility (M)	**-3804**	**7648**	-0.042	-3982	7965	0.029	-3888	7799	0.008
SD+BE	**-3880**	7812	-0.035	-3984	7968	0.030	-3891	**7799**	0.003
SD+M	**-3794**	**7636**	-0.049	-3926	7852	0.017	-3869	7760	0.001
BE+M	**-3819**	**7680**	-0.026	-3957	7912	0.027	-3861	7745	-0.004
SD+BE+M (Full)	**-3809**	**7670**	-0.040	-3908	7812	0.020	-3858	7737	-0.001
**Boston**									
Core	**-2035**	**4090**	-0.005	-2205	4415	0.127	-2076	4183	0.036
Social-disorganization (SD)	**-2019**	**4055**	-0.004	-2040	4080	0.018	-2033	4077	0.019
Built environment (BE)	**-2015**	**4050**	-0.034	-2136	4273	0.060	-2025	4080	0.009
Mobility (M)	**-2000**	**4025**	-0.026	-2087	4171	0.036	-2019	4074	-0.015
SD+BE	**-1987**	**4002**	-0.043	-2030	4060	0.027	-1996	4011	-0.008
SD+M	**-1973**	**3978**	-0.031	-2009	4016	0.013	-1991	4000	-0.012
BE+M	**-1990**	**4005**	-0.033	-2076	4151	0.036	-2007	4050	-0.007
SD+BE+M (Full)	**-1957**	**3943**	-0.039	-1993	3985	0.012	-1976	3974	-0.010
**LA**									
Core	**-9665**	**19398**	0.032	-10392	20820	0.092	-9950	19962	0.048
Social-disorganization (SD)	**-9528**	**19122**	0.004	-9953	19917	0.046	-9677	19378	0.011
Built environment (BE)	**-9629**	**19320**	0.005	-10185	20368	0.072	-9731	19477	0.013
Mobility (M)	**-9570**	**19183**	0.018	-10274	20549	0.080	-9858	19741	0.041
SD+BE	**-9509**	**19067**	-0.010	-9930	19860	0.044	-9608	19240	0.001
SD+M	**-9467**	**18990**	-0.002	-9872	19740	0.041	-9625	19265	0.012
BE+M	**-9585**	**19226**	0.011	-10193	20385	0.077	-9712	19432	0.018
SD+BE+M (Full)	**-9453**	**18956**	-0.010	-9892	19777	0.044	-9582	19182	0.001

Table S7: Results of two alternative connectivity matrices fusing together spatial information (distance) and human mobility. Mixed1 uses a connectivity matrix that balances the contribution of the distance and the number of trips between areas, while Mixed2 models two separate random effects. Chicago does not have mobility information, thus it was not possible to use the mobility matrix. We evaluate the models through LOO, DIC, and MC_p.

Supplementary Note 7 Spatial model decomposition

The fit of our model can be decomposed in fixed effects, which are the input variables, random effects, which are the unexplained variance through spatial auto-correlation, and residuals, which are the errors of the model.

From Figure S12 D, Figure S13 D, Figure S14 D, and Figure S15 D we do not observe any clear spatial pattern on the residuals, confirming that the BSF model is easing the spatial auto-correlation as expected.

The observation of the random effects can help on locating local spatial effects that are not considered in the fixed effects. In Bogotá, the model suggests that significant unexplained variance is present near the touristic and dangerous neighbourhood La Candelaria, and near the populous district of Engativá (see Figure S13). In Boston, the area near the Franklin park indicates missing local factors (see Figure S12). In Los Angeles, unexplained variance seems to be tied to places with a large amount of people, namely the international airport and the UCLA campus (see SI Figure S14). Finally in Chicago missing variables are suggested near the prison and the southern area (see Figure S15).

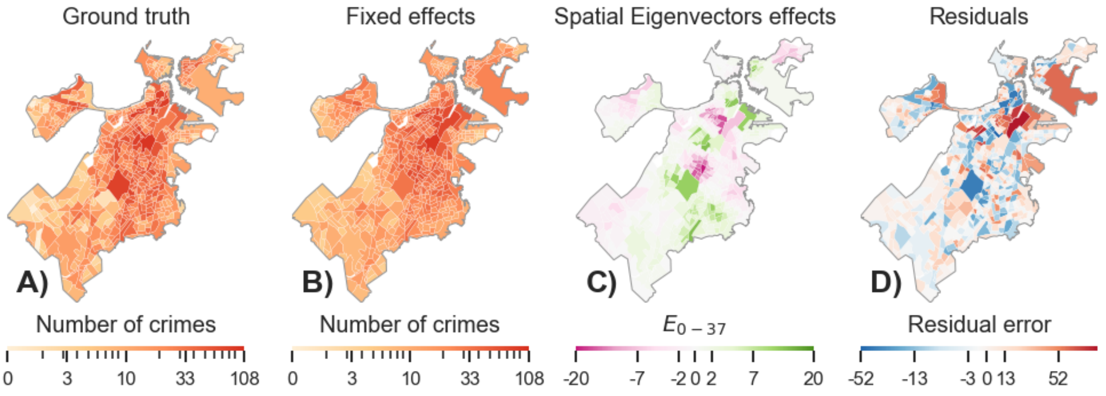

Figure S12: Decomposition of the ground truth in fixed, random and residuals effects in Boston.

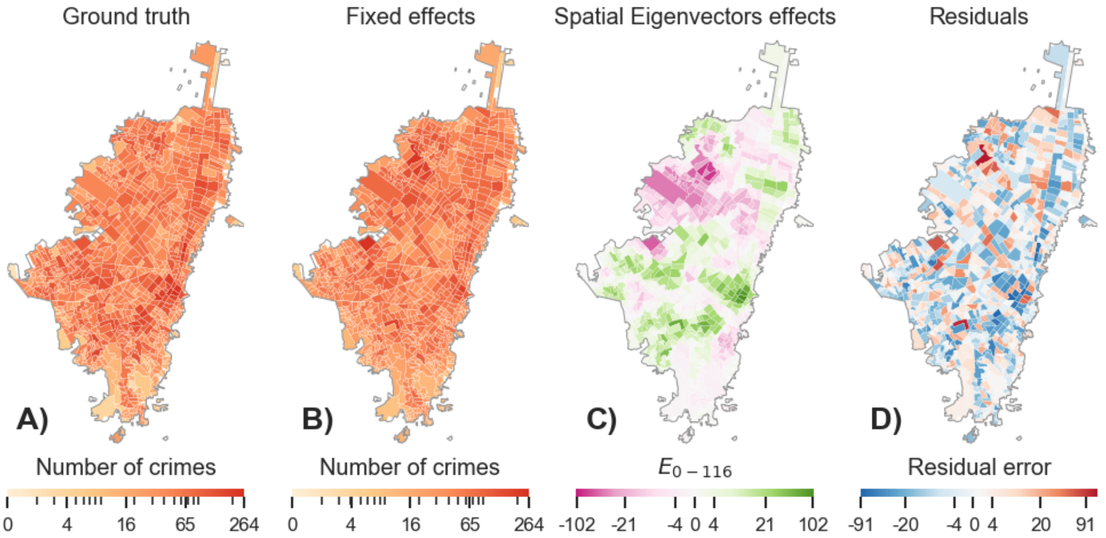

Figure S13: Decomposition of the ground truth in fixed, random and residuals effects in Bogotá.

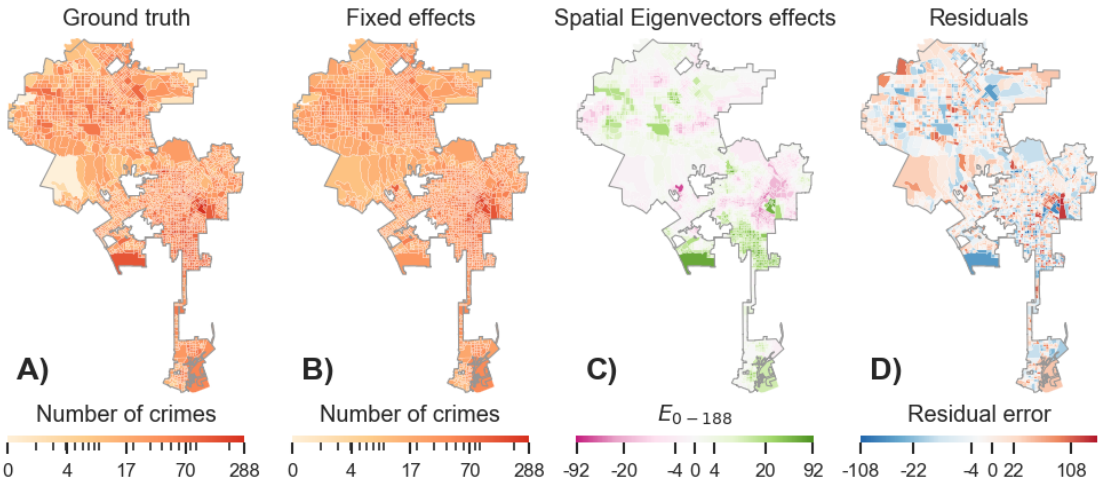

Figure S14: Decomposition of the ground truth in fixed, random and residuals effects in Los Angeles.

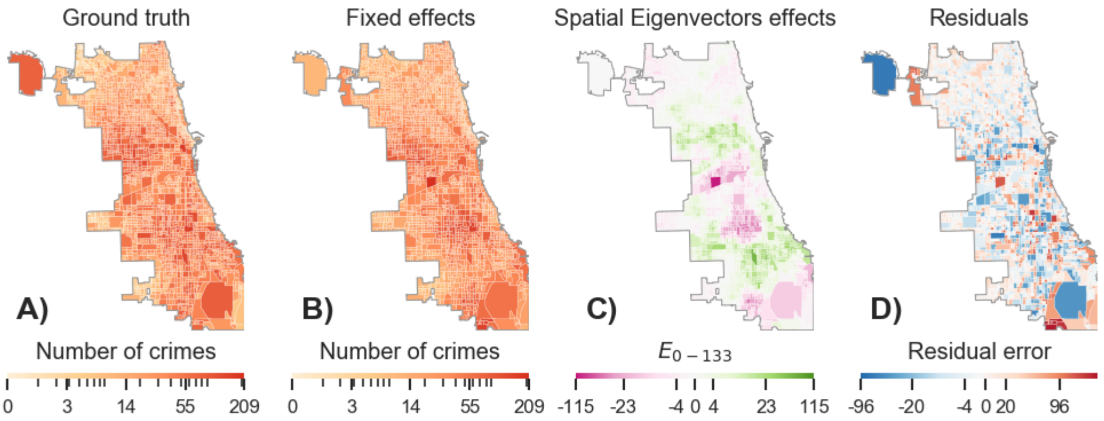

Figure S15: Decomposition of the ground truth in fixed, random and residuals effects in Chicago.

Supplementary Note 8 Improvement analysis

Figure S16, Figure S17, Figure S18 and Figure S19 show the improvement of each model against the Core model, and some reference variables for each city.

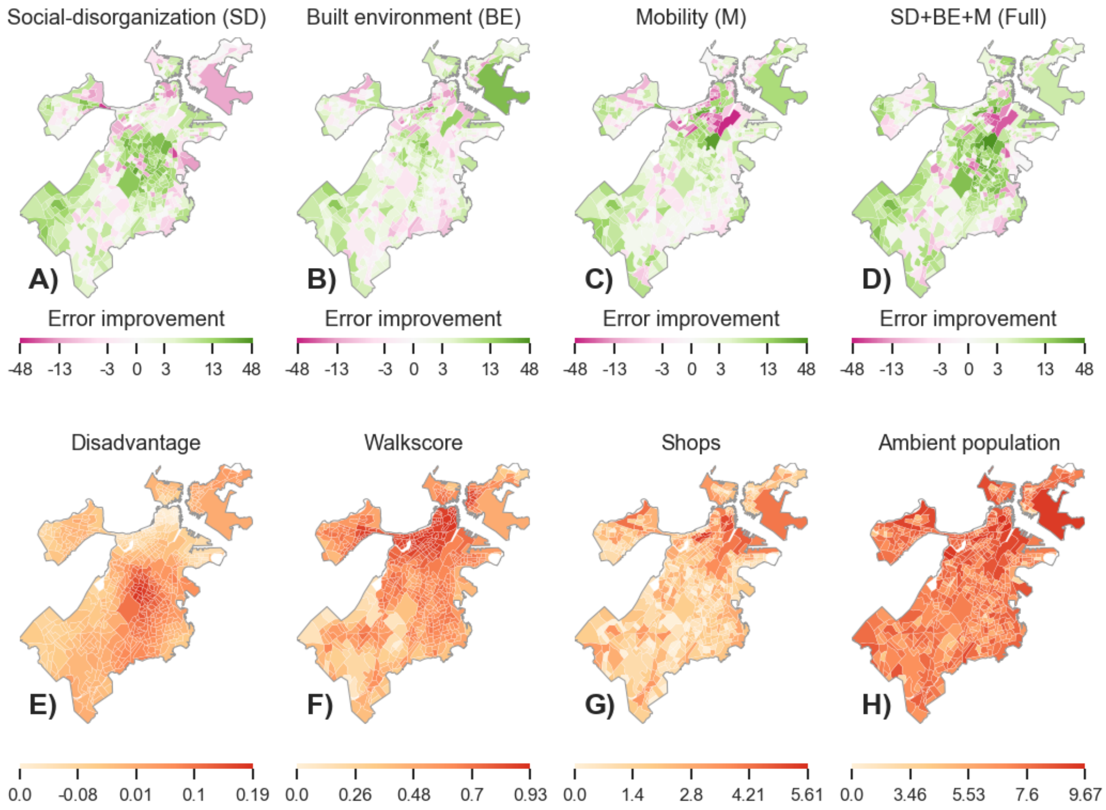

Figure S16: Model improvements in Boston. It can be seen that SD model improves the prediction from the core model almost everywhere but especially in disadvantaged areas, while the BE model seems to better improve the prediction near the airport and peripheral areas. The mobility model seems to improve but it also generates a strong outlier that performs poorly near the city centre. Finally, the Full model outperforms the core model and the other models almost everywhere. It keeps failing in some areas due to mobility information.

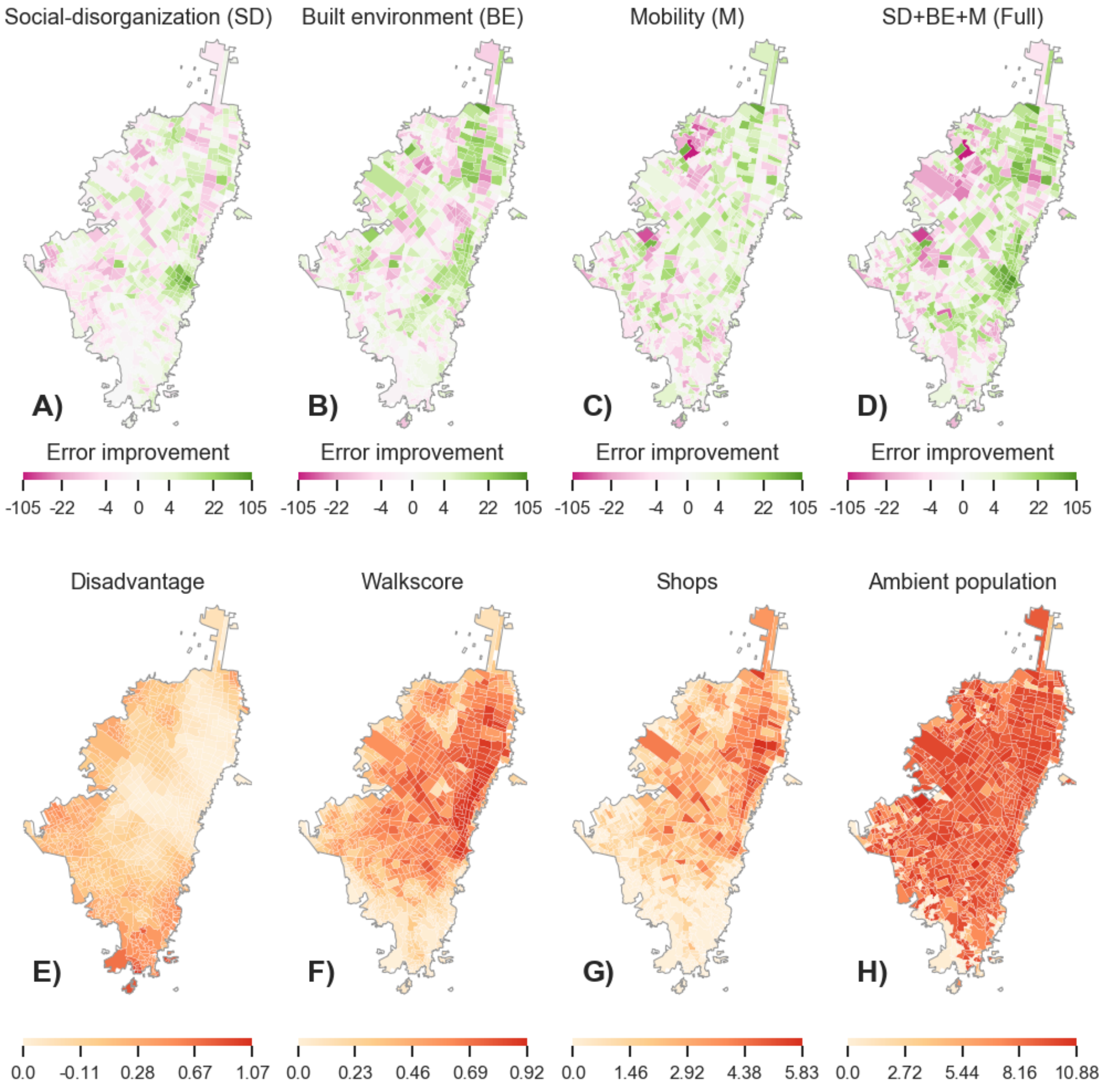

Figure S17: Model improvements in Bogotá. It can be seen that SD model improves the prediction from the core model very slightly, while the BE model seems to better improve the prediction near the richer part of the city and in areas with high number of shops. The mobility model seems to improve but it also generates a strong outlier that performs poorly near the "Engativa", a populous neighbourhood in Bogotá. Finally, the Full model outperforms the core model and the other models almost everywhere. It keeps failing in some areas due to mobility information.

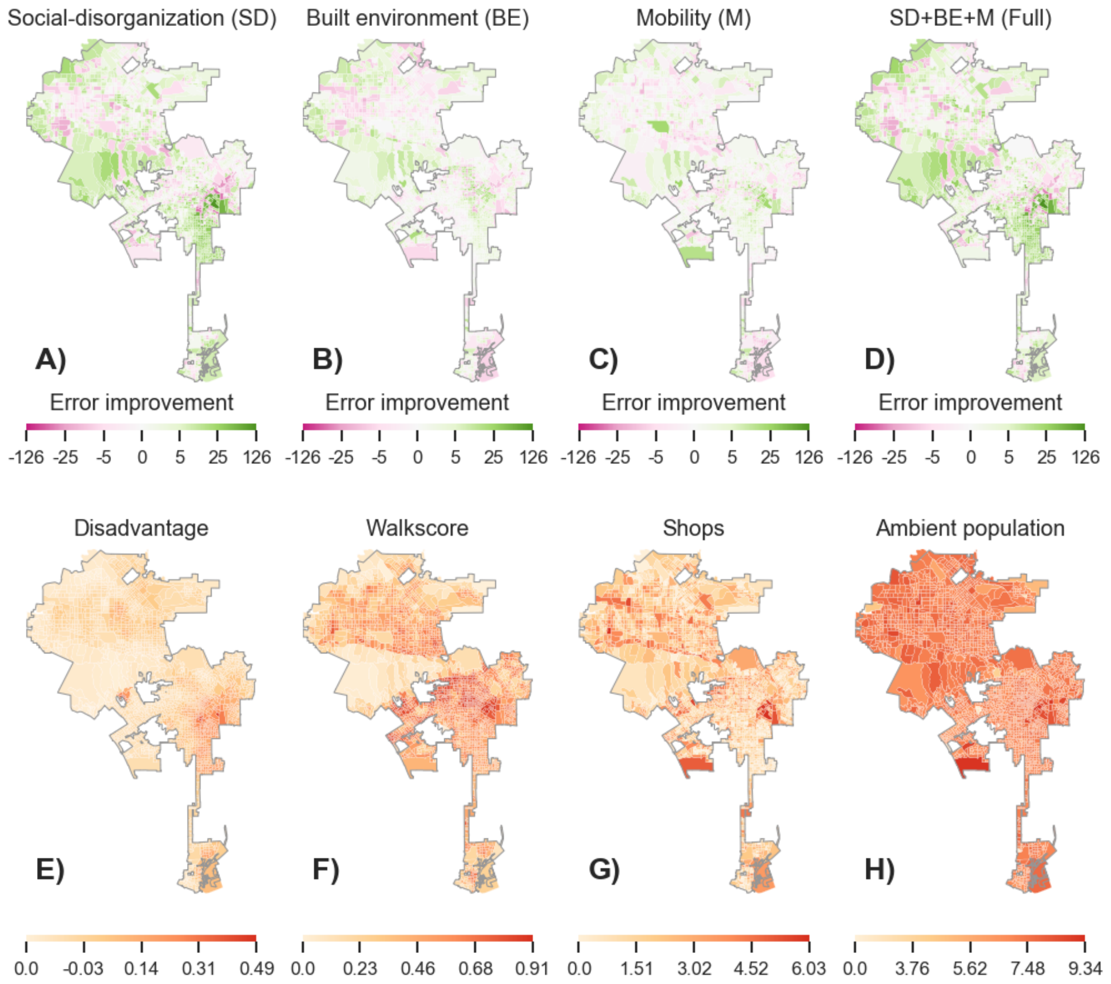

Figure S18: Model improvements in Los Angeles. It can be seen that SD model improves the prediction from the core model very consistently, especially in deprived areas, while the BE model seems to only slightly improve the prediction. The mobility model seems to improve especially in popular areas, such the airport. Finally, the Full model outperforms the core model and the other models almost everywhere.

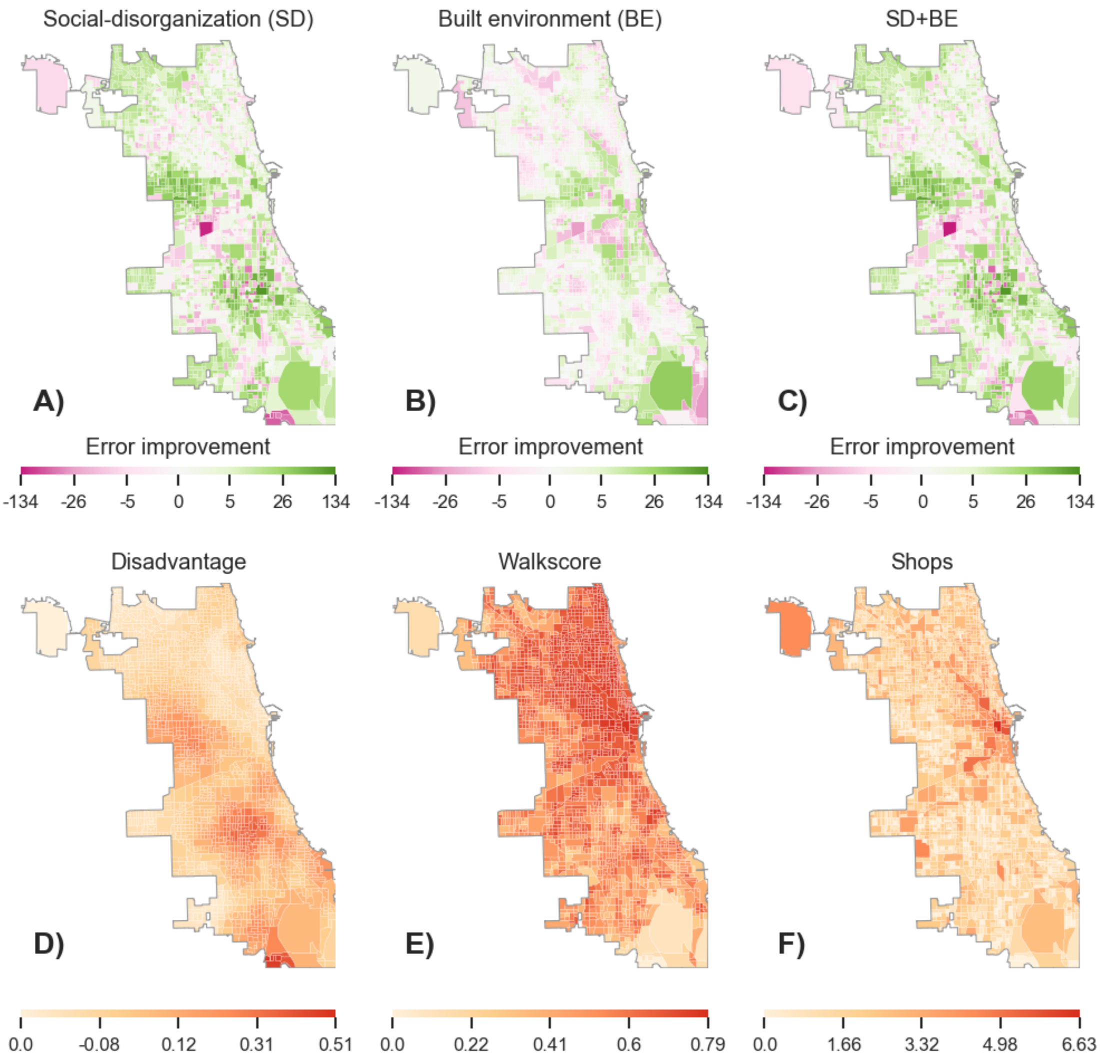

Figure S19: Model improvements in Chicago. Here, we note that we do not possess mobility information, so we compare the SD, BE and SD+BE models. It can be seen that SD model improves the prediction from the core model very consistently, especially in deprived areas, while the BE model seems to improve the prediction in southern Chicago. The SD+BE outperforms the core model and the other models almost everywhere.

Supplementary Note 9 Auto-correlation of features

Figure S20 shows how the features do not act with the same strength and direction in all cities.

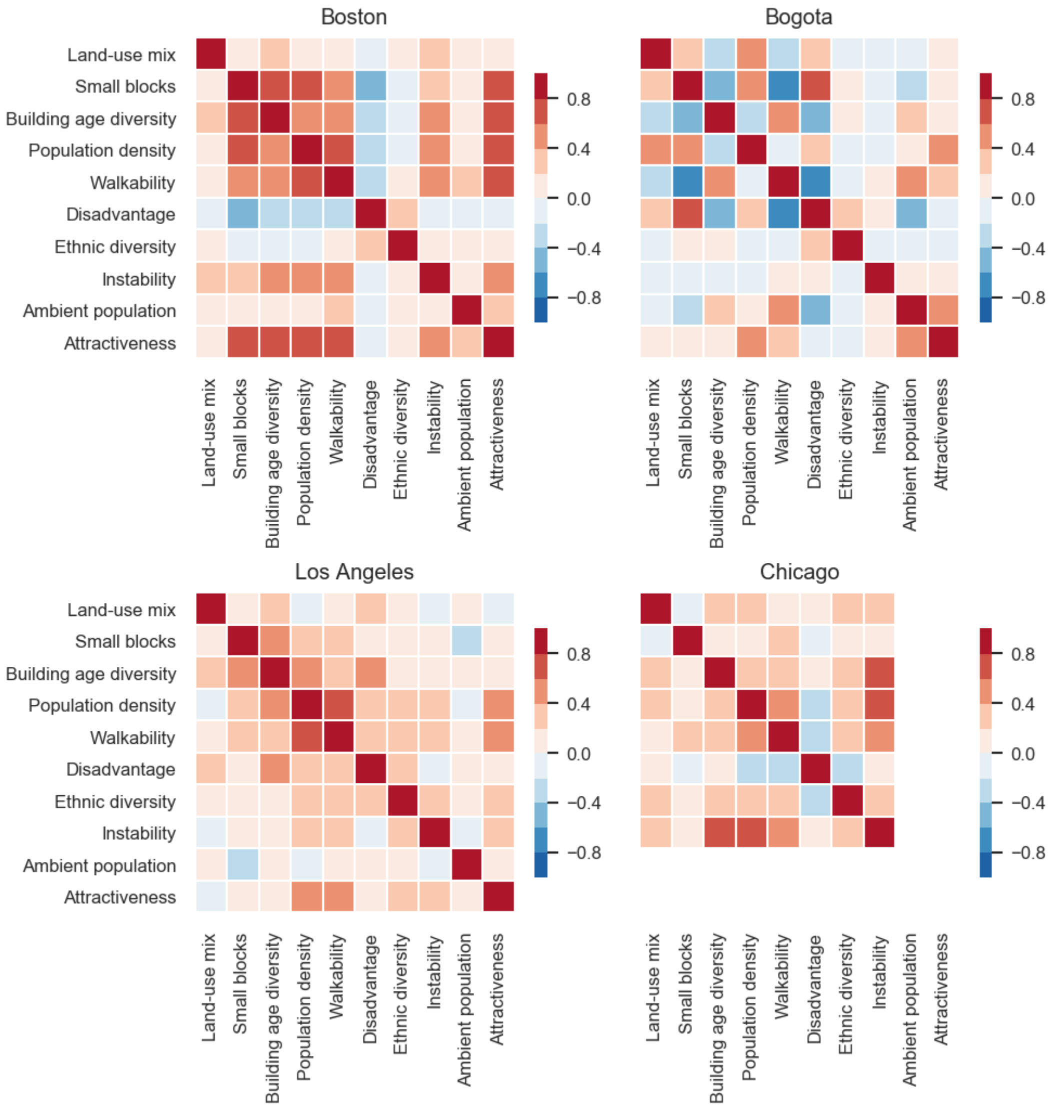

Figure S20: Correlation of features in the different cities.

Supplementary Note 10 The minimal model

Table S8 shows the results of the minimal model, which employs only the features that play the same role in all the cities. Results show that no minimal setting is better at

predicting crime in all cities.

City	Model	LOO	DIC	$\mathbf{R^2_m(R^2_c)}$	$\mathbf{MC_p}$
Bogotá	Minimal	-3872	7795	0.60 (0.66)	-0.046
	Full	**-3809**	**7670**	**0.70 (0.80)**	-0.040
Boston	Minimal	-1994	4014	0.64 (0.71)	-0.028
	Full	**-1957**	**3943**	**0.70 (0.75)**	-0.040
Los Angeles	Minimal	-9534	19128	0.49 (0.71)	-0.005
	Full	**-9453**	**18956**	**0.56 (0.73)**	0.003
Chicago	Minimal	-8009	16059	0.67 (0.78)	0.012
	Full	**-8001**	**16048**	**0.67 (0.78)**	0.003

Table S8: Results of the Full model and the minimal one, which exploits only those features that play the same role in all the cities.

Supplementary Note 11 Corehood tests

In criminology, the majority of works rely on administrative units, assuming that census units (and census tracts) could reflect the actual community boundaries [46]. However, the debate about the proper level of aggregation when estimating the neighbourhood effect is very active [79], even across different disciplines. Researchers have proposed to group neighboring areas into homogeneous contiguous regions [22, 27, 73], multi-scale comparisons [48], and continuously overlapping units [47, 54]. All these approaches have to deal with the well known Modifiable Area Unit Problem (MAUP) [66, 65, 36, 71] that arises from arbitrarily determining areal units that can be aggregated and disaggregated to form different areas with different size. Grid-based approaches, in particular, suffer the additional problem of combining different information available at different spatial aggregations, also called Change Of Support Problem (COSP) [40]. For example, in grid-based approaches, one has to decide how to spread the information available at irregular polygon (e.g. population at the block level) to a regular cell. Most of the works assume an unrealistic homogeneity in all directions and split or aggregate data through polygons intersections [72]. We tried to avoid COSP problems by using corehoods.

Given that there are no technical solutions to the MAUP issues [79, 46] one might suggest to avoid at all any aggregation [80]. However, researchers have to wrestle between the *micro-scale is a better* approach with the aggregations that explain social processes [79]. Hipp *et al.* [46] argue that theory should be the guideline to choose the appropriate spatial unit, preferably moving towards geographically smaller units. Thus, we followed this approach. We proposed a new spatial unit called corehood. The idea of using overlapping units is not new [57, 47, 33], with one notable example called *egohoods* [47]. Differently from *egohoods* we analyze crime at micro-scale (i.e. blocks) and aggregate blocks only to define the neighbourhood context at which the block is exposed,

while *egohoods* continuosly aggregate all the variables, including crime. We control the spatial-autocorrelation with state of the art Bayesian Spatial Filtering model (BSF) [49], which also allows to estimate the highest probability density of all the parameters including the spatial auto-correlation, and we regularize also the multicollinearity with the Ridge penalty and QR decomposition.

The features at the corehood are not computed only at the core since the social-disorganization and urban planning theories assume the existence of neighbourhoods. These neighborhoods are believed to be the place where social cohesion and cooperation between neighbours influence crime activities [44, 75, 74, 75, 64, 50]. Neighbourhoods are often assumed to be bigger than the building and the block, and they are usually loosely defined, even by residents [30]. In our submitted manuscript, we tested for different sizes of corehoods (see Section Supplementary Note 11) as suggested by literature and found that our definition better fits crime activity.

However, we also test two assumptions. First, that the chosen size of the corehood (0.5 miles) is not the best. Second, that the corehood should not be used at all.

Table S9 shows the results of the Full model, and SD+BE model in Chicago, for different sizes of Corehood. From the results we can observe that the best size to infer the neighborhood effect is half a mile.

Table S10 shows instead the test where we do not compute all the proposed features (SD, BE, M) at the corehood, but at the core. We note that this test can also be interpreted as setting the corehood size equal to the size of the core. For the core models we use a binary connectivity matrix **C** representing the spatial contiguity of cores: $c_{i,j} = 1$ if i and j are contiguous. We evaluate the models with features through the LOO, DIC but also the R2 marginal (R^2_m, which highlights the portion of variance explained by the fixed effects. If the assumption of the neighborhood effect is correct, we would expect higher performance in the LOO and higher R2 marginal. Table S10 shows that the models with features computed at the corehood perform better than the models using SD, BE and M features only at the core.

**City**	**Model**	**Corehood size**	**LOO**	**DIC**	$\mathbf{R^2_m (R^2_c)}$	$\mathbf{MC_p}$
Bogotá	Full	0.5 miles	**-3809**	**7670**	0.70 (**0.80**)	-0.040
		1 mile	-3860	7739	**0.71** (0.77)	-0.005
Boston	Full	0.5 miles	**-1957**	**3943**	**0.70 (0.75)**	-0.040
		1 mile	-2026	4068	0.65 (0.65)	-0.011
Los Angeles	Full	0.5 miles	**-9453**	**18956**	0.56 (**0.73**)	0.003
		1 mile	-9644	19314	**0.57** (0.68)	-0.001
Chicago	SD+BE	0.5 miles	**-8001**	**16048**	**0.67 (0.78)**	0.003
		1 mile	-8066	16158	0.66 (0.76)	-0.016

Table S9: Results of the Full or SD+BE model with different sizes of Corehood.

Model	Corehood			Core		
	LOO	DIC	R^2_m	LOO	DIC	R^2_m
**Bogotá**						
Social-disorganization (SD)	**-3891**	**7833**	**0.57**	-4099	8201	0.56
Built environment (BE)	**-3881**	**7807**	**0.61**	-4078	8156	0.59
Mobility (M)	**-3803**	**7647**	**0.64**	-4107	8222	0.56
SD+BE	**-3881**	**7812**	**0.64**	-4056	8109	0.61
SD+M	**-3795**	**7634**	**0.66**	-4083	8171	0.58
BE+M	**-3819**	**7680**	**0.68**	-4066	8132	0.60
SD+BE+M (Full)	**-3809**	**7670**	**0.70**	-4046	8092	0.62
**Boston**						
Social-disorganization (SD)	**-2019**	**4057**	**0.55**	-2178	4356	0.33
Built environment (BE)	**-2015**	**4050**	0.36	-2170	4344	**0.36**
Mobility (M)	**-2000**	**4025**	**0.42**	-2192	4386	0.27
SD+BE	**-1987**	**4002**	**0.65**	-2147	4295	0.44
SD+M	**-1973**	**3978**	**0.67**	-2155	4313	0.39
BE+M	**-1990**	**4006**	**0.50**	-2151	4307	0.41
SD+BE+M (Full)	**-1957**	**3944**	**0.70**	-2126	4256	0.48
**LA**						
Social-disorganization (SD)	**-9528**	**19122**	**0.54**	-10421	20902	0.40
Built environment (BE)	**-9629**	**19320**	0.27	-10568	21236	**0.31**
Mobility (M)	**-9570**	**19183**	**0.24**	-10752	21592	0.17
SD+BE	**-9509**	**19068**	**0.56**	-10357	20816	0.43
SD+M	**-9467**	**18991**	**0.55**	-10417	20906	0.40
BE+M	**-9585**	**19227**	0.30	-10556	21221	**0.31**
SD+BE+M (Full)	**-9453**	**18957**	**0.56**	-10352	20795	0.43
**Chicago**						
Social-disorganization (SD)	**-8019**	**16087**	**0.66**	-8881	17772	0.45
Built environment (BE)	**-8370**	**16808**	0.21	-9190	18396	**0.26**
SD+BE	**-8002**	**16049**	**0.67**	-8784	17574	0.51

Table S10: Results of models with features computed at the corehood and core level. We evaluate the models through LOO, R^2_m, DIC.

Supplementary Note 12 Disentangled crime types

Table S11 shows an additional comparison on different crime types. We divided crime into violent and property crime, following the UCR classification. The property crimes emphasize "breaking and entering" and includes the offences of burglary, larceny-theft, motor vehicle theft, and arson. Violent crime is instead composed of four offences: murder and non-negligent manslaughter, rape, robbery, and aggravated assault. To be comparable with the main table in the manuscript, we evaluate the model through two descriptive metrics, namely R_m^2 and R_c^2, and one predictive metric (LOO).

From the aggregated results in Table S11 we see that our Full model best performs in predicting violent and property crimes in all cities but Bogota. In this city, the Full model best describes crime (i.e. R_m^2 is the highest, while R_c^2 is near the best performance), while it performs worse at LOO. This might be explained by the fact that to nowcast crime the model can rely on the spatial auto-correlation and thus it does not need explanatory variables through the fixed effects (i.e. corehood features). However, in order to describe the factors related to crime, fixed effects are important. Figure S21 and Figure S22 show the β coefficients for the Full model applied for Violent and Property crime respectively. By analysing the β coefficients of our model, a few differences can be noted. Overall, these figures shows that even for spatial predictions of disentangled criminal activity there seems not to be an universal theory of crime.

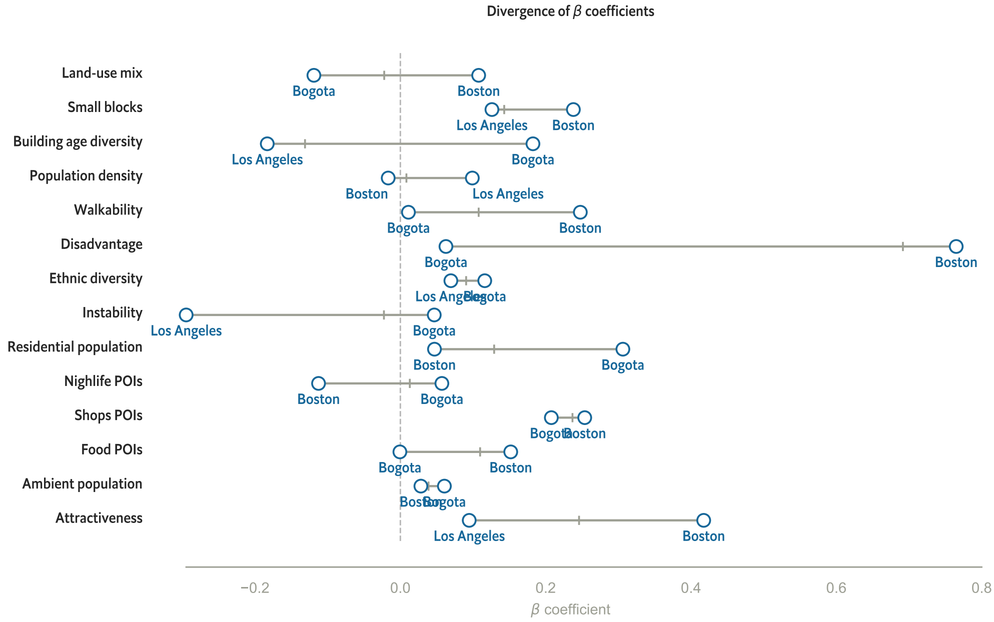

Figure S21: Generalized Linear Model's β coefficients on Violent crime. We highlight in blue the minimum and maximum coefficient for each feature.

Model	Bogota			Boston			Los Angeles			Chicago		
	LOO	R^2_m	R^2_c	LOO	R^2_m	R^2_c	LOO	R^2_m	R^2_c	LOO	R^2_m	R^2_c
**Violent crime**												
Core	-3731	0.45	0.73	-1674	0.20	0.71	-7656	0.13	0.77	-7171	0.08	0.71
SD	-3721	0.49	0.73	-1651	0.58	0.74	-7482	0.63	0.80	-6768	0.70	0.80
BE	-3717	0.56	0.74	-1653	0.35	0.73	-7515	0.34	0.80	-7148	0.17	0.71
M	-3653	0.55	0.78	-1664	0.42	0.72	-7590	0.22	0.78	-	-	-
SD+BE	-3717	0.59	0.74	-1623	0.69	0.78	-7414	0.69	0.82	**-6762**	**0.70**	**0.81**
SD+M	**-3638**	0.58	**0.78**	-1624	0.68	0.77	-7462	0.66	0.80	-	-	-
BE+M	-3671	0.62	0.77	-1648	0.48	0.74	-7488	0.36	0.80	-	-	-
SD+BE+M (Full)	-3659	**0.65**	0.77	**-1609**	**0.72**	**0.79**	**-7383**	**0.69**	**0.82**	-	-	-
**Property crime**												
Core	-2631	0.58	0.74	-1702	0.20	0.53	-8657	0.18	0.51	-7057	0.10	0.56
SD	-2630	0.62	0.74	-1706	0.44	0.53	-8593	0.33	0.54	-6869	0.47	0.64
BE	-2634	0.60	0.74	-1704	0.33	0.54	-8645	0.19	0.52	-7008	0.23	0.58
M	**-2591**	0.63	0.77	-1676	0.33	0.59	-8599	0.20	0.54	-	-	-
SD+BE	-2627	0.63	0.74	-1695	0.50	0.56	-8579	0.36	0.55	**-6848**	**0.51**	**0.65**
SD+M	-2593	0.66	0.77	-1669	0.52	0.60	-8552	0.33	0.56	-	-	-
BE+M	-2598	0.64	0.76	-1679	0.42	0.59	-8614	0.21	0.53	-	-	-
SD+BE+M (Full)	-2592	**0.67**	**0.77**	**-1668**	**0.55**	**0.61**	**-8537**	**0.36**	**0.57**	-	-	-

Table S11: Comparison on the different types of crime in all the cities, evaluated through LOO, R^2_m and R^2_c to have a consistent with the main manuscript.

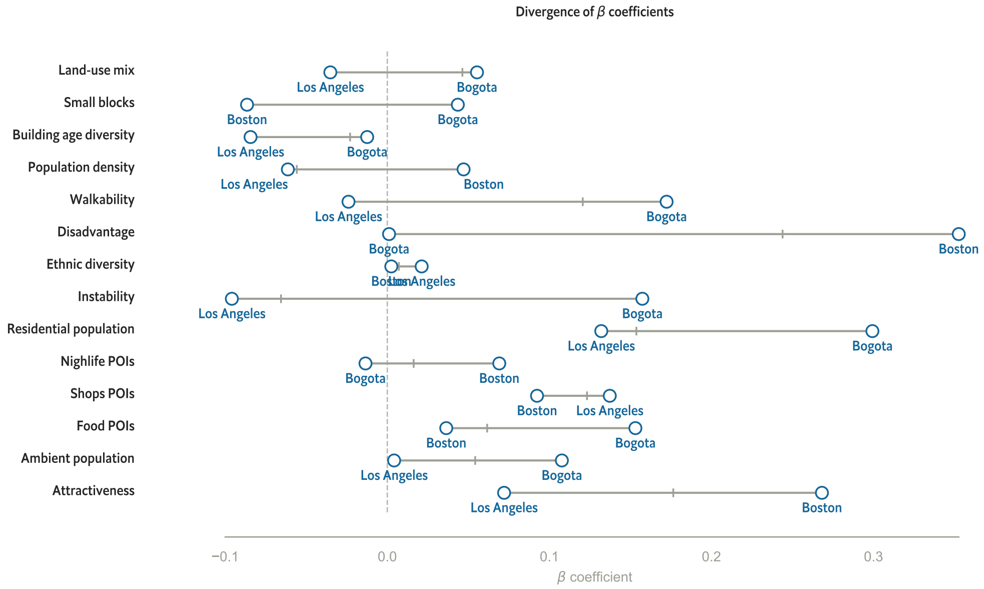

Figure S22: Generalized Linear Model's β coefficients on Property crime. We highlight in blue the minimum and maximum coefficient for each feature.

References

[1] Bogota buildings. https://www.ideca.gov.co/es/servicios/mapa-de-referencia/tabla-mapa-referencia?tid_1=All&title=&submit-b=Filtrar, (Last accessed June 21, 2020).

[2] Boston buildings. https://data.boston.gov/dataset/boston-buildings, (Last accessed June 21, 2020).

[3] Boston crime. https://data.boston.gov/dataset/crime-incident-reports-july-2012-august-2015-source-legacy-system, (Last accessed June 21, 2020).

[4] Boston landuse. https://data.boston.gov/dataset/parcels-2016-data-full, (Last accessed June 21, 2020).

[5] Boston maps open data site. http://bostonopendata-boston.opendata.arcgis.com/datasets/city-of-boston-boundary, (Last accessed June 21, 2020).

[6] Chicago boundaries. https://data.cityofchicago.org/Facilities-Geographic-Boundaries/Boundaries-Community-Areas-current-/cauq-8yn6.

[7] Chicago buildings. https://data.cityofchicago.org/Buildings/Building-Footprints-deprecated-August-2015-/qv97-3bvb, (Last accessed June 21, 2020).

[8] Chicago crime. https://data.cityofchicago.org/Public-Safety/Crimes-2001-to-present/ijzp-q8t2, (Last accessed June 21, 2020).

[9] Chicago landuse. https://datacatalog.cookcountyil.gov/GIS-Maps/ccgisdata-Parcel-2014/2m9h-cq6j, (Last accessed June 21, 2020).

[10] Cmap land use inventory. https://datahub.cmap.illinois.gov/group/land-use-inventories, (Last accessed June 21, 2020).

[11] Front seat walk score methodology. Technical report. Available online at http://pubs.cedeus.cl/omeka/files/original/b6fa690993d59007784a7a26804d42be.pdf. Last accessed on 3 January 2020, (Last accessed February 20, 2020).

[12] Ideca. https://www.ideca.gov.co/es/servicios/mapa-de-referencia/tabla-mapa-referencia?tid_1=All&title=&submit-b=Filtrar, (Last accessed June 21, 2020).

[13] La boundaries. https://data.lacity.org/A-Well-Run-City/Neighborhoods/ykhe-zspy/data, (Last accessed June 21, 2020).

[14] Los angeles buildings. https://egis3.lacounty.gov/dataportal/2016/11/03/countywide-building-outlines-2014-update-public-domain-release/, (Last accessed February 20, 2020).

[15] Los angeles crime. https://data.lacity.org/A-Safe-City/Crimes-2012-2015/s9rj-h3s6, (Last accessed February 20, 2020).

[16] Los angeles landuse. https://egis3.lacounty.gov/dataportal/2015/03/10/assessor-parcel/, (Last accessed February 20, 2020).

[17] Us census factfinder. https://factfinder.census.gov, (Last accessed June 21, 2020).

[18] Us census tiger. ftp://ftp2.census.gov/geo/tiger/TIGER2014/TABBLOCK/, (Last accessed June 21, 2020).

[19] M. A. Andresen. Crime measures and the spatial analysis of criminal activity. *British Journal of criminology*, 46(2):258–285, 2006.

[20] M. A. Andresen. The ambient population and crime analysis. *The Professional Geographer*, 63(2):193–212, 2011.

[21] N. Arnold, A. Thomas, L. Waller, and E. Conlon. Bayesian models for spatially correlated disease and exposure data. In *Bayesian Statistics 6: Proceedings of the Sixth Valencia International Meeting*, volume 6, page 131. Oxford University Press, 1999.

[22] R. M. Assunção, M. C. Neves, G. Câmara, and C. da Costa Freitas. Efficient regionalization techniques for socio-economic geographical units using minimum spanning trees. *International Journal of Geographical Information Science*, 20(7):797–811, 2006.

[23] H. Barbosa, M. Barthelemy, G. Ghoshal, C. R. James, M. Lenormand, T. Louail, R. Menezes, J. J. Ramasco, F. Simini, and M. Tomasini. Human mobility: Models and applications. *Physics Reports*, 734:1–74, 2018.

[24] V. D. Blondel, A. Decuyper, and G. Krings. A survey of results on mobile phone datasets analysis. *EPJ data science*, 4(1):10, 2015.

[25] R. Boivin and M. Felson. Crimes by visitors versus crimes by residents: The influence of visitor inflows. *Journal of Quantitative Criminology*, 34(2):465–480, 2018.

[26] California Department of Transportation (Caltrans). California household travel survey (CHTS). https://dot.ca.gov/programs/transportation-planning/economics-data-management/transportation-economics/ca-household-travel-survey. Last accessed 19 June 2020.

[27] C. Caminha, V. Furtado, T. H. Pequeno, C. Ponte, H. P. Melo, E. A. Oliveira, and J. S. Andrade Jr. Human mobility in large cities as a proxy for crime. *PloS one*, 12(2):e0171609, 2017.

[28] Y. Chun, D. A. Griffith, M. Lee, and P. Sinha. Eigenvector selection with stepwise regression techniques to construct eigenvector spatial filters. *Journal of Geographical Systems*, 18(1):67–85, 2016.

[29] S. Çolak, L. P. Alexander, B. G. Alvim, S. R. Mehndiratta, and M. C. González. Analyzing cell phone location data for urban travel: current methods, limitations, and opportunities. *Transportation Research Record*, 2526(1):126–135, 2015.

[30] C. J. Coulton, J. Korbin, T. Chan, and M. Su. Mapping residents' perceptions of neighborhood boundaries: a methodological note. *American journal of community psychology*, 29(2):371–383, 2001.

[31] S. D. de Movilidad. Informe de indicadores encuesta de movilidad de bogota 2011. `https://www.simur.gov.co/portal-simur/datos-del-sector/encuestas-de-movilidad/`, (Last accessed June 21, 2020).

[32] M. De Nadai, A. Cardoso, A. Lima, B. Lepri, and N. Oliver. Strategies and limitations in app usage and human mobility. *Scientific reports*, 9(1):1–9, 2019.

[33] M. De Nadai and B. Lepri. The economic value of neighborhoods: Predicting real estate prices from the urban environment. In *2018 IEEE 5th International Conference on Data Science and Advanced Analytics (DSAA)*, pages 323–330. IEEE, 2018.

[34] L. Frank, M. Bradley, S. Kavage, J. Chapman, and T. K. Lawton. Urban form, travel time, and cost relationships with tour complexity and mode choice. *Transportation*, 35(1):37–54, 2008.

[35] N. J. Garber and L. A. Hoel. *Traffic and highway engineering*. Cengage Learning, 2014.

[36] C. E. Gehlke and K. Biehl. Certain effects of grouping upon the size of the correlation coefficient in census tract material. *Journal of the American Statistical Association*, 29(185A):169–170, 1934.

[37] A. Gelman, J. B. Carlin, H. S. Stern, D. B. Dunson, A. Vehtari, and D. B. Rubin. *Bayesian data analysis*. CRC press, 2013.

[38] A. Gelman et al. Prior distributions for variance parameters in hierarchical models (comment on article by browne and draper). *Bayesian analysis*, 1(3):515–534, 2006.

[39] M. C. Gonzalez, C. A. Hidalgo, and A.-L. Barabasi. Understanding individual human mobility patterns. *nature*, 453(7196):779–782, 2008.

[40] C. A. Gotway and L. J. Young. Combining incompatible spatial data. *Journal of the American Statistical Association*, 97(458):632–648, 2002.

[41] C. Graif, B. N. Freelin, Y.-H. Kuo, H. Wang, Z. Li, and D. Kifer. Network spillovers and neighborhood crime: A computational statistics analysis of employment-based networks of neighborhoods. *Justice Quarterly*, pages 1–31, 2019.

[42] C. Graif, A. S. Gladfelter, and S. A. Matthews. Urban poverty and neighborhood effects on crime: Incorporating spatial and network perspectives. *Sociology compass*, 8(9):1140–1155, 2014.

[43] C. Graif, A. Lungeanu, and A. M. Yetter. Neighborhood isolation in chicago: Violent crime effects on structural isolation and homophily in inter-neighborhood commuting networks. *Social Networks*, 2017.

[44] C. Graif and R. J. Sampson. Spatial Heterogeneity in the Effects of Immigration and Diversity on Neighborhood Homicide Rates. *Homicide Studies*, 13(3):242–260, 2009.

[45] D. A. Griffith and P. R. Peres-Neto. Spatial modeling in ecology: the flexibility of eigenfunction spatial analyses. *Ecology*, 87(10):2603–2613, 2006.

[46] J. R. Hipp. Block, tract, and levels of aggregation: Neighborhood structure and crime and disorder as a case in point. *American Sociological Review*, 72(5):659–680, 2007.

[47] J. R. Hipp and A. Boessen. Egohoods as waves washing across the city: A new measure of "neighborhoods". *Criminology*, 51(2):287–327, 2013.

[48] J. R. Hipp, Y.-A. Kim, and J. C. Wo. Micro-scale, meso-scale, macro-scale, and temporal scale: Comparing the relative importance for robbery risk in new york city. *Justice Quarterly*, pages 1–25, 2020.

[49] J. Hughes. Spatial regression and the bayesian filter. *arXiv preprint arXiv:1706.04651*, 2017.

[50] J. Jacobs. *The death and life of great American cities.* Vintage, 1961.

[51] S. Jiang, G. A. Fiore, Y. Yang, J. Ferreira Jr, E. Frazzoli, and M. C. González. A review of urban computing for mobile phone traces: current methods, challenges and opportunities. In *Proceedings of the 2nd ACM SIGKDD international workshop on Urban Computing*, pages 1–9, 2013.

[52] S. Jiang, Y. Yang, S. Gupta, D. Veneziano, S. Athavale, and M. C. González. The TimeGeo modeling framework for urban mobility without travel surveys. *PNAS*, 113(37):E5370–E5378, 2016.

[53] C. Kadar, R. R. Brüngger, and I. Pletikosa. Measuring ambient population from location-based social networks to describe urban crime. In *International Conference on Social Informatics*, pages 521–535. Springer, 2017.

[54] Y.-A. Kim and J. R. Hipp. Street egohood: An alternative perspective of measuring neighborhood and spatial patterns of crime. *Journal of Quantitative Criminology*, 36(1):29–66, 2020.

[55] G. Krings, F. Calabrese, C. Ratti, and V. D. Blondel. Urban gravity: a model for inter-city telecommunication flows. *Journal of Statistical Mechanics: Theory and Experiment*, 2009(07):L07003, 2009.

[56] L. J. Krivo, R. A. Byron, C. A. Calder, R. D. Peterson, C. R. Browning, M.-P. Kwan, and J. Y. Lee. Patterns of local segregation: do they matter for neighborhood crime? *Social science research*, 54:303–318, 2015.

[57] I. Lee, S. Jung, J. Lee, and E. Macdonald. Street crime prediction model based on the physical characteristics of a streetscape: Analysis of streets in low-rise housing areas in south korea. *Environment and Planning B: Urban Analytics and City Science*, 46(5):862–879, 2019.

[58] G. Lin and T. Zhang. Loglinear residual tests of moran's i autocorrelation and their applications to kentucky breast cancer data. *Geographical Analysis*, 39(3):293–310, 2007.

[59] N. Malleson and M. A. Andresen. Spatio-temporal crime hotspots and the ambient population. *Crime science*, 4(1):10, 2015.

[60] L. W. Mburu and M. Helbich. Crime Risk Estimation with a Commuter-Harmonized Ambient Population. *Annals of the American Association of Geographers*, 106(4):804–818, 2016.

[61] M. G. McNally. The four step model. *Handbook of transport modelling*, 1:35–41, 2000.

[62] P. A. Moran. The interpretation of statistical maps. *Journal of the Royal Statistical Society. Series B (Methodological)*, 10(2):243–251, 1948.

[63] D. Murakami and D. A. Griffith. Eigenvector spatial filtering for large data sets: fixed and random effects approaches. *Geographical Analysis*, 51(1):23–49, 2019.

[64] O. Newman. *Defensible space.* Macmillan New York, 1972.

[65] S. Openshaw. The modifiable areal unit problem. *Quantitative geography: A British view*, pages 60–69, 1981.

[66] S. Openshow. A million or so correlation coefficients, three experiments on the modifiable areal unit problem. *Statistical applications in the spatial science*, pages 127–144, 1979.

[67] D. W. Osgood. Poisson-based regression analysis of aggregate crime rates. *Journal of quantitative criminology*, 16(1):21–43, 2000.

[68] L. Pappalardo, D. Pedreschi, Z. Smoreda, and F. Giannotti. Using big data to study the link between human mobility and socio-economic development. In *2015 IEEE International Conference on Big Data (Big Data)*, pages 871–878. IEEE, 2015.

[69] R. Patuelli, D. A. Griffith, M. Tiefelsdorf, and P. Nijkamp. Spatial filtering methods for tracing space-time developments in an open regional system: Experiments with german unemployment data. *Societies in Motion: Innovation, Migration and Regional Transformation*, pages 247–68, 2012.

[70] R. F. Potthoff. Homogeneity, potthoff-whittinghill tests of. *Encyclopedia of Statistical Sciences*, 2006.

[71] W. S. Robinson. Ecological correlations and the behavior of individuals. *International journal of epidemiology*, 38(2):337–341, 2009.

[72] G. Rosser, T. Davies, K. J. Bowers, S. D. Johnson, and T. Cheng. Predictive crime mapping: Arbitrary grids or street networks? *Journal of Quantitative Criminology*, 33(3):569–594, 2017.

[73] H. D. Rozenfeld, D. Rybski, X. Gabaix, and H. A. Makse. The area and population of cities: New insights from a different perspective on cities. *American Economic Review*, 101(5):2205–25, 2011.

[74] R. J. Sampson. Neighborhoods and Violent Crime: A Multilevel Study of Collective Efficacy. *Science*, 277(5328):918–924, 1997.

[75] R. J. Sampson and W. B. Groves. Community structure and crime: Testing social-disorganization theory. *American Journal of Sociology*, 94(4):774–802, 1989.

[76] D. J. Spiegelhalter, N. G. Best, B. P. Carlin, and A. Van Der Linde. Bayesian measures of model complexity and fit. *Journal of the Royal Statistical Society: Series B (Statistical Methodology)*, 64(4):583–639, 2002.

[77] D. J. Spiegelhalter, N. G. Best, B. P. Carlin, and A. Van der Linde. The deviance information criterion: 12 years on. *Journal of the Royal Statistical Society: Series B (Statistical Methodology)*, 76(3):485–493, 2014.

[78] M. Tiefelsdorf and D. A. Griffith. Semiparametric filtering of spatial autocorrelation: the eigenvector approach. *Environment and Planning A*, 39(5):1193–1221, 2007.

[79] G. E. Tita and S. M. Radil. Making space for theory: the challenges of theorizing space and place for spatial analysis in criminology. *Journal of Quantitative Criminology*, 26(4):467–479, 2010.

[80] W. R. Tobler. Frame independent spatial analysis. *Accuracy of spatial databases*, pages 115–122, 1989.

[81] J. L. Toole, S. Colak, B. Sturt, L. P. Alexander, A. Evsukoff, and M. C. González. The path most traveled: Travel demand estimation using big data resources. *Transportation Research Part C: Emerging Technologies*, 58:162–177, 2015.

[82] F. H. A. US Department of Transportation. National household travel survey. `http://nhts.ornl.gov`. Last accessed 19 June 2020.

[83] A. Vehtari, A. Gelman, and J. Gabry. Practical bayesian model evaluation using leave-one-out cross-validation and waic. *Statistics and computing*, 27(5):1413–1432, 2017.

[84] H. Wang and Z. Li. Region representation learning via mobility flow. In *Proceedings of the 2017 ACM on Conference on Information and Knowledge Management*, pages 237–246, 2017.

[85] Y. Zheng and X. Xie. Learning travel recommendations from user-generated gps traces. *ACM Transactions on Intelligent Systems and Technology (TIST)*, 2(1):1–29, 2011.
